# Diet and temperature interactively impact brown adipose tissue gene regulation controlled by DNA methylation

**DOI:** 10.1016/j.molmet.2025.102315

**Published:** 2026-01-01

**Authors:** Tobias Hagemann, Anne Hoffmann, Kerstin Rohde-Zimmermann, Helen Broghammer, Lucas Massier, Peter Kovacs, Michael Stumvoll, Matthias Blüher, John T. Heiker, Juliane Weiner

**Affiliations:** 1 Helmholtz Institute for Metabolic, Obesity and Vascular Research (HI-MAG) of the Helmholtz Zentrum München at the University of Leipzig and University Hospital Leipzig, Leipzig, Germany; 2 Department of Medicine H7, Karolinska Institute, Stockholm, Sweden; 3 Leipzig University Medical Center, Divisions of Endocrinology and Nephrology, University of Leipzig, Leipzig, Germany

**Keywords:** brown adipose tissue, DNA methylation, High fat diet, Cold exposure, Epigenetics, Thermogenesis

## Abstract

**Background:**

Controlling brown adipose tissue (BAT) plasticity offers potential for novel obesity therapies. DNA methylation is closely linked to thermogenic and metabolic pathways and thereby influences BAT function. How metabolic state and cold exposure interact to shape methylation-dependent BAT gene regulation was investigated.

**Methods:**

Five-week-old mice were fed either chow for 11 weeks (lean) or high-fat diet for 22 weeks to induce obesity (DIO), after which cold exposure was applied for seven days. BAT transcriptomes (RNAseq) and methylomes (RRBS) were generated, and differentially methylated and expressed genes (DMEGs) showing metabolic state–dependent cold responses were identified. Pathway enrichment, epigenetic regulator screening, and transcription factor (TF) motif analyses were performed. DNA methylation was experimentally modulated *in vitro* to validate selected gene expression responses.

**Results:**

A total of 1,364 differentially expressed genes (DEGs) were uniquely affected by the interaction of metabolic state and cold, with most downregulated in DIO mice. Sixty-five DMEGs (4 % of DEGs) showed metabolic state–specific responses to cold. In DIO mice, DMEGs were enriched in pathways associated with mitochondrial dysfunction, altered lipid metabolism, neuroendocrine signaling, and stress responses. Several epigenetic regulators, including *Tet2, Dnmt3a,* and *Apobec1*, exhibited metabolic state- and cold-dependent expression, and TF-motif analyses highlighted roles for AhrArnt and Foxn1. In vitro assays confirmed that DNA methylation influences expression of thermogenic genes.

**Conclusion:**

These findings provide the first evidence that the epigenetic cold response of BAT differs by metabolic condition. BAT remodeling is shaped by coordinated transcriptional and epigenetic mechanisms integrating environmental and metabolic cues.

## Highlights

• Obese mice show impaired cold adaptation, with weight loss and persistent hypothermia compared to lean controls.
• Cold activates BAT thermogenic genes, but transcriptional responses are quantitatively reduced in obesity.
• Epigenetic remodeling of BAT is limited in obesity, with fewer DMEGs and increased gene-body methylation.
• Obesity redirects cold-induced pathways toward stress and diabetogenic signaling and away from browning.
• Core methylation regulators respond to cold, but adaptive epigenetic reprogramming is constrained in obesity.

## Graphical abstract

Mice were housed under cold exposure (8 °C) or thermoneutrality (30 °C) and fed either chow (lean) or high-fat diet (HFD; diet-induced obese (DIO)). RNA sequencing (RNAseq) and reduced representation bisulfite sequencing (RRBS) was performed on brown adipose tissue (BAT) which provide the fundament for the identification of differentially methylated positions (DMP) and regions (DMR) as well as differentially expressed genes (DEG) in three models: *COLD*_*lean*_, comparing 8° versus 30 °C mice on chow diet; *COLD*_*DIO*_, comparing 8° versus 30 °C mice on HFD diet and Δ*COLD*, comparing *COLD*_*lean*_ versus *COLD*_*DIO*_. Differentially methylated and expressed genes (DMEGs) were identified for all comparisons, based on DMPs within DMRs that significantly correlated with differentially expressed genes (DEGs; the heatmap shows a representative example). DMEGs were further characterized. First, using a published single cell dataset (Shamsi et al., 2023, https://doi.org/10.1038/s42003-023-05140-2), the distribution of DMEGs across cell types was analyzed. For further characterization, DMEGS were categorized into similarly (among *COLD*_*lean*_ and *COLD*_*DIO*)_ and uniquely (*COLD*_*lean*_, *COLD*_*DIO*__and_ Δ*COLD*) regulated. Pathway enrichment analyses were performed and to identify differentially regulated epigenetic regulators among the DMEGs, the EpiFactor Database (https://epifactors.autosome.org) was used. A TF-binding site motive enrichment analyses (https://jaspar.elixir.no)was applied to add information of most enriched Transcription Factors (TF). Finally, to gain functional insights of DNA methylation changes on selected DMEG candidates, cell cultures of immortalized brown adipocytes and primary brown adipocytes were treated with 5′aza-2′-deoxycytidine (demethylation) or S-adenosylmethionine (upregulation of methylation), following gene expression analyses. The figure was created with BioRender.

**Figure:**
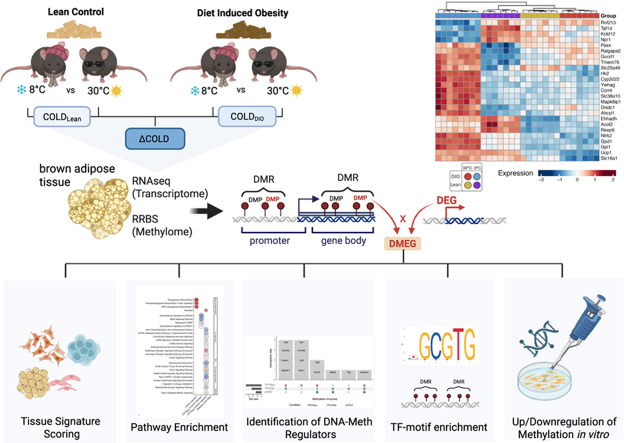

## 1 Introduction

Brown adipose tissue (BAT) is a specialized fat tissue that is rich in mitochondria and promotes non-shivering thermogenesis by expressing the *uncoupling protein 1 (UCP1).* Unlike white adipose tissue, it burns calories to produce heat and therefore plays a key role in energy regulation and controlling metabolic health. Increasing energy expenditure by BAT activation is an intriguing therapeutic approach to combat the overwhelming obesity pandemic, either alone or to complement the current pharmacotherapy that mainly addresses energy intake based on the incretin-mimetic poly-agonist class of drugs [1]. With this in mind it is not surprising that a lot of research was conducted to understand the molecular underpinnings of BAT regulation specifically addressing environmental cues. Cold exposure is the most powerful inducer of BAT activation leading to the upregulation of thermogenic gene program and adrenergic receptor-mediated activation of lipolysis and metabolism. BAT activation also occurs post-prandially, especially after acute overfeeding, to trigger diet-induced thermogenesis [2,3]. However, this compensatory component of energy-expenditure is impaired during chronic overfeeding, a phenomenon that was termed adaptive thermogenesis, and is believed to further drive weight gain and obesity [2,4,5].

In any case the mechanistical switch enabling the high plasticity of BAT changing from passive thermoneutral to active thermogenic states may be regulated by epigenetic mechanisms that are affected by, e.g. temperature and diet [6]. Indeed, studies have shown changes in DNA methylation for key BAT regulatory genes such as *Ucp1, PR domain containing 16* (*Prdm16*) or *PPARG coactivator 1 alpha* (*Ppargc1a*), altering their expression level and impacting BAT function [7,8]. Moreover, during the development of precursor cells into major brown adipocytes, DNA methylation patterns change and lineage commitment towards a thermogenic adipocyte is epigenetically controlled [8]. The major relevance of the ambient temperature in this context has just recently been implied, as cold exposure of parents at conception is related to increased brown adipogenesis and thermogenesis along with reduced obesity risk in the offspring [9]. Mechanistically, sperm DNA methylation is altered in response to cold exposure which leads to adaptations in BAT activity of the offspring [10,11]. In addition, DNA methylation in key genes of thermogenesis and fatty acid oxidation, such as *acetyl-CoA acyltransferase 2* (*Acaa2*) and *acyl-CoA synthetase long chain family member 1* (*Acsl1*), is increased in BAT of offsprings from diet-induced obese mice fed a high fat diet [12]. These studies strongly imply the relevance of cold, diet and obesity on the transgenerational modulation of BAT function. However, these environmental cues, either alone or in combination, also trigger and control BAT plasticity during adulthood. Diet-induced obese rats with a blockade in afferent vagal nerve signaling triggered by high-fat diet showed a reduced BAT activation after cold exposure [13]. The relevance of diet for regulating DNA methylation in later life comes with nutritional compounds such as folate or methionine as critical components of the one-carbon metabolism, which are classically enriched in vegetable-rich diets but diminished in high fat diets.

However, the interaction of especially cold exposure and obesity on BAT's epigenetic machinery is less understood. In this study, we aimed to address DNA methylation alterations in response to temperature and metabolic state, and link this to changes in gene expression of BAT in mice on a genome-wide level. We explored the interactive impact of metabolic state and cold exposure on the epigenetic regulation of BAT gene expression. Using a stringent bioinformatic workflow we focused on differentially methylated and expressed genes (DMEGs) by taking forward only candidates whose differential methylation signals correlates to the gene expression level in order to provide only top functional relevant candidates. Providing a profound overview over the impact of DNA methylation on genes expression affected by cold and metabolic state is of strong relevance to understand adult BAT plasticity and adaptive thermogenesis and provides the fundament for new obesity treatment strategies.

## 2 Material and methods

### 2.1 Animal studies

Male C57BL/6NTac mice (Taconic Bioscience, Lille Skensved, Denmark) were housed in pathogen-free facilities at 23 °C on a 12 h light/dark cycle at the Sächsische Inkubator für Klinische Translation (SIKT), Leipzig. Starting from five weeks of age, mice were either fed a standard chow diet (EV153, 3.3 % from fat, Ssniff, Soest, Germany) or a high fat diet (HFD, E15742, 60 kJ% from fat, Ssniff) with ad libitum access to food and water. For the purposes of subsequent analyses, chow-fed mice are referred to as lean, whereas HFD-fed mice, which became obese after 17 weeks, are referred to as diet-induced obese (DIO). At 11 (lean) or 22 (DIO) weeks of age, mice were adapted to single housing in rodent climate chambers (MKKL1200, Flohr Instruments, Netherlands) for 5 days, before randomization into four groups (each N = 8 mice) and housed either at thermoneutrality (30 °C) or in the cold (8 °C) for 7 days. After a 4-hour fasting period, body weight and rectal temperature were measured. BAT and tail surface temperatures were measured by thermal imaging (VarioCAM®, InfraTec, Dresden, Germany). Mice were sacrificed and tissue samples of intrascapular brown adipose tissue (BAT) were collected, snap frozen in liquid nitrogen and stored at −80 °C before further analyses. All animal experiments were approved by the local authorities of the Free State of Saxony, Germany (Landesdirektion Leipzig: TVV51/20, T09/21), as recommended by the responsible local animal ethics review board.

### 2.2 RNA sequencing of brown adipose tissue

RNA from BAT was isolated using RNeasy Lipid Tissue Mini kit (Qiagen, Hilden, Germany) as specified by the manufacturer. RNA sequencing (RNAseq) was performed by the core unit DNA-Technologien, Medical Faculty Leipzig. 50 ng of total RNA were depleted of ribosomal RNA using the NEBNext rRNA Depletion Kit v2 (NEB, USA) according to the manufacturer's instructions. Depleted RNA was transcribed using SuperScript IV reverse transcriptase (ThermoFisher Scientific, USA) for 2 h at 55 °C. After second strand synthesis (TargetAmp kit, EPICENTRE, USA), the DNA was fragmented using the Illumina Tagment DNA TDE1 Enzyme and Buffer Kits, which fragments DNA and inserts partial sequencing adapter sequences. Final PCR amplification of the libraries was done using KAPA HiFi HotStart Library Amplification Kit with unique dual indexing by IDT for Illumina Nextera DNA Unique Dual Indexes Sets. The barcoded libraries were purified and quantified using Qubit Fluorometric Quantification (ThermoFisher Scientific, USA). Size distribution of the library DNA was analyzed using the FragmentAnalyzer (Agilent, USA). Sequencing of 2x150 bp was performed with NovaSeq sequencer (Illumina, USA) according to the manufacturer's instructions, with a read depth of 20 million reads per sample.

### 2.3 Reduced representation bisulfite sequencing in brown adipose tissue

Genomic DNA from BAT samples were isolated using the DNeasy blood and tissue kit according to the manufacturer's instructions (Qiagen, Hilden, Germany). Reduced representation bisulfite sequencing (RRBS) library preparation was performed on 100 ng DNA using the Zymo-Seq RRBS Library Kit (Zymo Research, Freiburg, Germany) by the core unit DNA-Technologien, Medical Faculty Leipzig. Briefly, DNA was digested with 20U/μl MspI for 4 h followed by RRBS adapter ligation using 400U/μl T4 DNA ligase and gap filling with 2U/μl Taq DNA polymerase and 10 mM 5-methylcytosine dNTP Mix. Bisulfite conversion was performed with lightning conversion reagent, and the final samples were amplified using 5 μM of the unique dual index Primer Set (indexes 1–12) supplied by the manufacturer. Quality control was performed on the Bioanalyzer (Illumina, USA) prior paired-end sequencing on the NovaSeq6000 platform (Illumina, USA) were 20 million reads per sample were obtained.

### 2.4 RNAseq data preprocessing

Raw RNAseq reads were quality-filtered and adapter-trimmed using Trimmomatic (v0.39) [14], discarding reads shorter than 36 bp. Afterwards, reads were aligned to GRCm38.p6 reference assembly using STAR (v2.7.8a) [15], allowing for a maximum of 50 multi-mapped reads. Gene counts were quantified with featureCounts from the subread software package (v2.0.1) [16] with multimappings counted by fractions.

### 2.5 Differential gene expression analysis

Transcripts without gene annotation and genes with a total count sum <10 were removed resulting in 18 k genes for subsequent gene expression analysis. Variance stabilization transformation (VST) was applied to the filtered gene count matrix using the R package DESeq2 (v1.42.1) [17]. Principal component analysis (PCA) was conducted to examine clustering according to metabolic state (lean, DIO) and temperature (8 °C, 30 °C) groups based on gene expression profiles. Three samples were identified as outliers which did not cluster in their respective group according to the first two principal components and were subsequently removed from the analysis. This resulted in group sizes of 7–8 mice reflecting all diet–temperature combinations (N_*total*_ = 29; N_*DIO*_30°C_ = 7; N_*DIO*_8°C_ = 7; N_*lean*_30°C_ = 7; N_*lean*_8°C_ = 8). Differentially expressed genes (DEGs) were identified using DESeq2. Three comparisons were conducted to assess: i) the cold response of lean mice (8 °C versus 30 °C; *COLD*_*lean*_), ii) the cold response of DIO mice (8 °C versus 30 °C; *COLD*_*DIO*_), and iii) metabolic state-dependent differences in the cold response using an interaction model (*ΔCOLD*) based on a comparison between *COLD*_*DIO*_ versus *COLD*_*lean*_. DEGs with a false discovery rate (FDR) < 0.05 were considered statistically significant. DEG lists from each contrast were subsequently cross-referenced with entries in the EpiFactors Database (v2.0, https://epifactors.autosome.org, accessed on September 10, 2024) to identify metabolic state-specific methylation regulators.

### 2.6 RBBS data processing

Raw RRBS reads from N = 29 mice (N_*DIO*_30°C_ = 7; N_*DIO*_8°C_ = 7; N_*lean*_30°C_ = 7; N_*lean*_8°C_ = 8) were processed with the nextflow (v 23.04.3) [18] pipeline nf-core/methylseq (v2.4.0) [19] using the parameters *--genome GRCm38 --non_directional --rrbs --skip_deduplication* and *--cytosine_report*. In brief, quality filtering and adapter trimming of raw RRBS fastq files was conducted with trimgalore (v0.6.7) [20] before running sequence alignments with Bismark (v0.24.0) [21]. Afterwards, methylation signals are called with the Bismark methylation extractor script including the build-in *coverage2cytosine* module for a genome-wide cytosine methylation report which served as input for downstream analysis. Sex chromosomes were excluded.

### 2.7 Differential methylation analysis

To identify differentially methylated positions (DMPs) and differentially methylated regions (DMRs), differential methylation analysis was conducted for *COLD*_*lean*_, *COLD*_*DIO*_, and *ΔCOLD*, utilizing beta values and the R packages limma (v3.54.0) [22] and RnBeads (v2.16.0) [23] with parameters filtering.low.coverage.masking = TRUE, filtering.high.coverage.outliers = TRUE and filtering.snp = “any”. Based on their chromosomal position and respective annotation, individual methylations can be contextualized within gene bodies or promoter regions (1,500 bases upstream and 500 bases downstream of the transcription start site), both of which were considered in our analysis. Only DMPs with |log_2_ fold change (FC)| >1 and p < 0.05 were retained for downstream analysis.

### 2.8 Multi-omics integration of methylation and transcriptome data

To identify genes in which DNA methylation changes are functionally linked to transcriptional regulation, both differential expression (DEGs) and methylation (DMRs, DMPs) profiles were jointly analyzed. The R package biomaRt (v2.58.2) [24] was used to gather gene loci information for DEGs and DMRs. Beta methylation values were transformed to M-values using RnBeads. For each DEG, only DMRs located within the corresponding gene and promotor region were selected. M-values of all DMPs within these DMRs were identified using the bedR (v1.0.7) [25] and the GenomicRanges (v1.54.1) [26] R packages. Pearson correlation was then applied to find associations between DEG expression values and M-values of each DMR-intersecting DMP using the multtest.cor function from RVAideMemoire (v0.9-83-7) [27]. Finally, DEGs (|log2 FC| > 0.5, FDR <0.05) were classified as differentially methylated and expressed genes (DMEGs) if at least one DMR-intersecting DMP (|log2 FC| > 1, p < 0.05) showed a correlation (|r| > 0.5, p < 0.05) with the gene's expression. If a DMEG is associated to both promoter and gene body region, it was assigned to the DMR with the highest significance. This step was parallelized with the foreach package (v1.5.2) [28]. For subsequent analysis steps, the set of DMEGs were categorized based on gene expression signals into similarly regulated DMEGs between metabolic states or uniquely regulated (*COLD*_*lean*_ and *COLD*_*DIO*_) and *ΔCOLD*.

#### 2.8.1 Pathway and regulator profiling of DMEGs

These DMEG groups were subject to functional analysis with the use of QIAGEN Ingenuity Pathway Analysis software (QIAGEN Inc., https://digitalinsights.qiagen.com/IPA, Winter Release Q4 2024) [29] covering the catalog for canonical pathways and upstream regulators. The database query was limited within the IPA software to relationships with adipose tissue or adipocytes in mice that are experimentally observed. Enrichments are reported with p after Bonferroni-Holm (BH) adjustment and activation z-scores. Enrichments with BH corrected p < 0.05 are considered significant.

#### 2.8.2 Transcription factor motif enrichment analysis

Enrichment analysis of transcription factor (TF) binding sites (TFBS) was conducted for similarly or uniquely regulated DMEGs with the R package motifTestR (v1.4.2) [30]. Only DMEGs located within promoter regions were retained. For each DMEG, the methylated locus was extended 20 nt upstream and downstream, and the corresponding DNA sequence was extracted from the masked mouse reference genome (mm10, GRCm38.p6) [31]. To construct a background distribution, promoter sequences of identical length were sampled randomly from the mm10 genome. This random sampling was repeated for n = 5,000 iterations using the makeRMRanges function, yielding a robust background set. Position-weight matrices (PWMs) for mouse TFBS were retrieved from JASPAR 2024 database [32] using MotifDB (v1.50.0) [33]. The testMotifEnrich function was used to evaluate motif enrichments in each DMEG set against the generated background. A quasipoisson regression model was applied, requiring a minimum motif-matching score of 80 %. To assess metabolic state-specific effects, enrichment of motifs in DMEGs uniquely regulated in *COLD*_*DIO*_ was tested using the *COLD*_*lean*_ DMEGs as a physiological background. This comparison employed a one-sided hypergeometric test. Motifs with p < 0.05 in either analysis were retained for downstream visualization.

### 2.9 *In vitro* validation experiments

#### 2.9.1 Immortalized and primary brown adipocytes

Immortalized (imBA [34]) and primary brown adipocytes were cultured and differentiated as previously described [35]. At day 10, cells were harvested and subjected to further analyses.

#### 2.9.2 *In vitro* manipulation of cellular DNA methylation levels

To analyze the effect of DNA methylation status on gene expression in imBA and primary brown adipocytes, pre-adipocytes were treated with indicated concentrations of 5′aza-2′-deoxycytidine (Aza) for 48 h and subsequently differentiated [35]. Differentiated adipocytes at day 6 were treated with indicated concentrations of S-adenosylmethionine (SAM, New England Biolabs) for 48 h. Differentiated adipocytes were harvested and subjected to further analysis.

#### 2.9.3 Quantitative real-time-PCR (qPCR)

RNA from imBA and human primary SAT cells was isolated using RNeasy Lipid Tissue Mini kit. qPCR was performed using the LightCycler System LC480 and LightCycler-DNA Master SYBR Green I Kit (Roche, Mannheim, Germany). Adipocyte gene expression of candidate genes was calculated by ΔΔCT method and normalized to *Nono* or *36b4* levels as indicated. Primer sequences are listed in (Suppl. Table 1).

### 2.10 Statistical analysis

Analyses were performed under R version 4.3.2, except for mouse phenotyping data that was analyzed using GraphPad Prism 10 (GraphPad, San Diego, CA, USA). Statistical tests are stated in the figure legends. If not stated otherwise, adj. p < 0.05 were considered statistically significant.

## 3 Results

### 3.1 Phenotyping of lean and diet-induced obese mice held at thermoneutrality or exposed to cold

**Figure 1:**
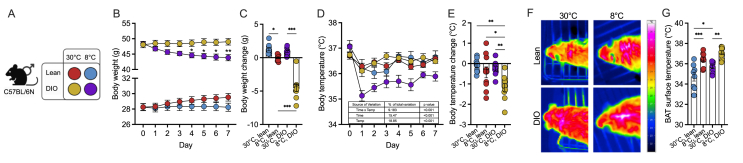
**Phenotyping of lean and diet-induced obese mice housed at 30°C or 8°C.** (A) Schematic overview and corresponding color code of the cohort mouse groups stratified by metabolic state (lean (chow-fed mice), diet-induced obese (DIO; HFD-fed mice)) and environmental (thermoneutrality versus cold) conditions. (B) Body weight development and (C) body weight change in lean and DIO mice housed at thermoneutrality (30 °C) or in the cold (8 °C) for 7 days. Body temperature (D) and temperature change (E) in lean and DIO mice housed at thermoneutrality (30 °C) or in the cold (8 °C) for 7 days. (F) Thermal images from brown adipose tissue (BAT) and (G) BAT surface temperature in lean and DIO mice housed at thermoneutrality (30 °C) or in the cold (8 °C) for 7 days. N = 7–8 per condition. (B, D) Statistical significance was evaluated by two-way ANOVA with uncorrected Fischer's LSD, (E) Šídák's post-hoc test, (C, G) or one-way ANOVA with Tukey's post-hoc test. p < 0.05 (∗), p < 0.01 (∗∗), p < 0.001 (∗∗∗). Scale bar: 100 μm.

Groups of 8 mice per metabolic state (lean chow-fed or DIO HFD-fed) were held at thermoneutrality (30 °C) or in the cold (8 °C) for 7 days (Figure 1A). Prior to the temperature challenge, mice were held at 23 °C, and DIO mice presenting significantly higher body weights compared to lean mice (48.2 g versus 28.3 g, Figure 1B). In the cold, lean mice maintained a steady body weight, while DIO mice lost about 10 % body weight (Figure 1B,C). All mice in the cold showed a significant drop in body temperature during the first days, but this loss in temperature was significantly higher in DIO mice (−0.5 °C versus −2 °C after d1, Figure 1D). After 7 days, lean mice recovered their body temperature to normal levels, while DIO mice still had significantly lower temperatures (−1 °C) (Figure 1D,E). Under thermoneutrality, DIO mice had significantly higher BAT surface temperatures, consistent with increased metabolic state-induced thermogenesis, but BAT surface temperatures were not different after cold exposure (Figure 1F,G). Extended phenotyping data is presented in Suppl. Figure 1.

### 3.2 Obesity impairs core transcriptome remodeling of BAT in response to cold

**Figure 2:**
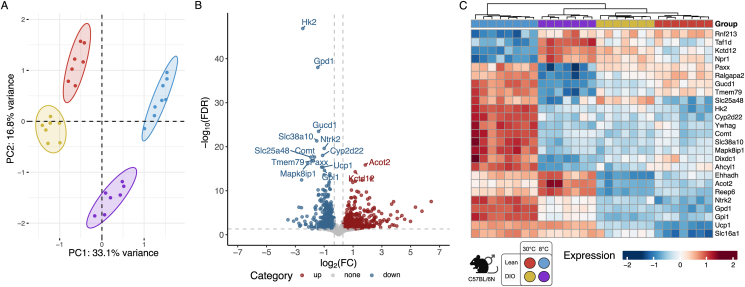
**RNA sequencing-based gene expression data revealse distinct clusters of treatment groups.** (A) Principal component analysis separates temperatures and metabolic states. All considered subgroups reveal distinct expression profiles and cluster according to the first two principal components. (B) Volcano plot highlights most significantly (false discovery rate (FDR) < 0.05) regulated genes under Δ*COLD* condition. (C) Heatmap of expression values shows that the subgroups can be clearly separated by the top significant regulated genes. Abbreviations: DIO: diet-induced obese; PC: principal component.

Gene expression data was used from all mice samples that also had methylation signal data available. After removal of outliers, N = 29 samples (N_*DIO*_30°C_ = 7; N_*DIO*_8°C_ = 7; N_*lean*_30°C_ = 7; N_*lean*_8°C_ = 8) remained for subsequent differential gene expression and methylation analysis. Analysis of VST normalized RNAseq expression counts shows clear clustering of all metabolic states (lean, DIO)- and temperature (8 °C, 30 °C)-specific subgroups in the principal component analysis (Figure 2A), providing a solid basis for subsequent differential gene expression analysis. Moreover, temperature groups are separated by principal component 1 describing 33 % of variance while metabolic state groups are rather distinguished by principal component 2 covering 16.8 % of total variance demonstrating an overall stronger impact of temperature compared to metabolic state on overall gene expression patterns. DIO mice are older compared to lean mice. The age gap of 11 weeks could potentially interfere and explain a variance in the principal component analyses which was not addressed here.

To dissect BAT's physiological response to cold in normal-weight control mice, we compared lean mice held in the cold (8 °C) with those held at thermoneutrality (30 °C) for one week. We will refer to this condition pair as *COLD*_*lean*_. Additionally, to assess the effects of obesity on cold-induced BAT activation, we compared DIO mice held in the cold (8 °C) with mice held at thermoneutrality (30 °C) for one week. This comparison will be referred to as *COLD*_*DIO*_. Differential gene expression analysis (FDR <0.05) resulted in 7,089 DEGs for *COLD*_*lean*_ (Suppl. Figure 2A, B; Suppl. Table 2) and 5,249 DEGs for *COLD*_*DIO*_ (Suppl. Figure 2C, D; Suppl. Table 3) condition pairs. Reflecting the dominating effect of temperature on BAT activity, the majority of the significant DEGs overlap between *COLD*_*lean*_ and *COLD*_*DIO*_ conditions are regulated similarly (N = 3,786; *COLD*_*lean*_: 53 %; *COLD*_*DIO*_: 72 %). Furthermore, 45 % and 26 % are specifically regulated in *COLD*_*lean*_ and *COLD*_*DIO*_, respectively, while a total of 92 DEGs are contrarily regulated (Suppl. Figure 3A).

To benchmark our *COLD*_*lean*_ results with previously published data, we overlapped our DEGs with those reported by Taylor et al. (2024) [36], who performed a comparable study in lean chow-fed male mice exposed to severe cold (8 °C) versus thermoneutrality (28 °C) (Suppl. Figure 4; Suppl. Table 2). We found that 80 % of our upregulated and 52 % of our downregulated genes overlapped with their DEGs, demonstrating a strong concordance across both directions in these independent analyses.

Additionally, to investigate how metabolic state affects BAT activation in response to cold exposure, we employed an interaction model (Δ*COLD*) contrasting *COLD*_*DIO*_ and *COLD*_*lean*_. This approach enabled the identification of metabolic state-dependent differences in the cold-induced transcriptional program, beyond effects attributable to individual factors alone [37]. Although an age gap of 11 weeks is indicated, lean or DIO mice are in a comparable age-range regarding BAT function and diet-independent body weight development [[38], [39], [40], [41]]. In total, we observed 1,364 genes (FDR <0.05) which are differentially expressed between *COLD*_*DIO*_ and *COLD*_*lean*_ (Figure 2B,C; Suppl. Table 4). Since the most significant DEGs are downregulated in DIO compared to lean mice under cold exposure, this suggests that obesity impairs the thermogenic activation of BAT, potentially reducing its capacity for energy expenditure.

### 3.3 Associations of gene expression and methylation signals underscore the influence of metabolic state on gene regulatory mechanisms

To explore the associative effects between transcriptomics and epigenomics, we correlated all expression signals of DEGs with DMPs found within the DMRs of the corresponding genes. This approach allowed us to define a set of differentially methylated and expressed genes (DMEGs), using specific criteria: a Pearson correlation coefficient greater than ± 0.5 (p < 0.05), DEGs with a log_2_ FC greater than ± 0.5 (FDR <0.05), and DMPs with a log_2_ FC greater than ± 1 (p < 0.05). Importantly, we set a higher FC threshold for DMPs and focused on DMEGs located in both the promoter and gene body regions, ensuring that our analysis captures the most relevant regulatory elements influencing gene expression. To verify our findings, we analyzed the effect of DNA-demethylation (using Aza) or hypermethylation (using SAM) on the expression of selected genes in mouse adipocytes *in vitro* (Suppl. Figure 5). The observed effects of DNA methylation and the manipulation on gene expression were in line with the results of our analysis correlating gene expression and methylation and confirmed epigenetic control of gene expression in adipocytes.

**Figure 3:**
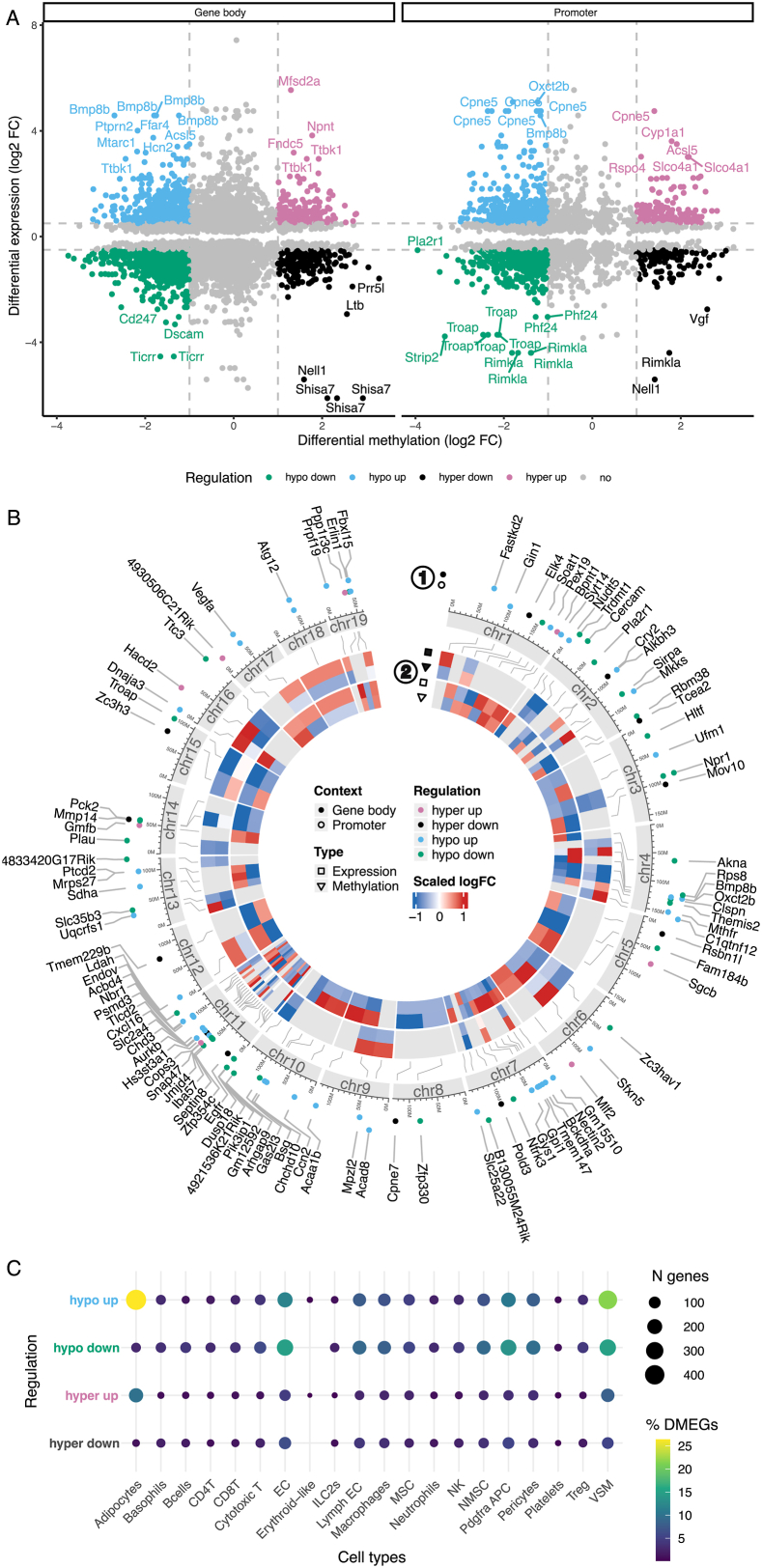
**Multi-layered insights into DMEGs under *COLD*_*lean*_ condition.** (A) The scatter plot displays the log_2_ fold change (FC) in gene expression and DNA methylation signals for all differentially methylated and expressed genes (DMEGs) in both gene body and promoter contexts under the *COLD*_*lean*_ condition. Colors indicate the direction of regulation. DMEGs with absolute log_2_ FC > 0.5 in expression and > 1 in methylation signals are highlighted, and labels are given for those DMEGs in the 5 % and 95 % quantile of both expression and methylation log_2_ FC. (B) The circos visualization illustrates the DMEGs covering the top 100 significant differentially methylated regions (DMRs; ranked by log_2_ FC). This plot integrates multiple layers of information from outer to inner ring: Dot colors indicate the direction of regulation, while dot positions denote whether the DMR of the DMEG is located in the gene body or promoter context (1); a stylized ideogram of chromosomes provides a genomic reference frame; and a heatmap displays scaled log_2_ FC for both gene expression and DNA methylation signals in gene body and promoter contexts for each DMEG (2). (C) Overview of assigned cell types to the DMEGs, categorized as hypo up, hypo down, hyper up, and hyper down. The color scale reflects the percentage of DMEGs, and the size of the dots reflects the absolute number of DMEGs.

In the *COLD*_*lean*_ control comparison, we identified a total of 1,524 DMEGs, corresponding to 21.5 % of all DEGs. These DMEGs were associated with 3,142 distinct DMR-intersecting DMPs, reflecting that a single DMEG can be linked to multiple methylation sites. While the majority of DMEGs were associated with only one DMP, approximately 13 % exhibited expression signal correlating with four or more DMPs (Suppl. Figure 6A). Such multi-site associations may indicate regionally coordinated or more stable methylation patterns, potentially reflecting broader epigenetic modulation rather than isolated CpG effects. Further, the data showed a balanced distribution of DMPs of the DMEGs, with 55 % located within gene bodies and 45 % found in promoter regions (Figure 3A; Suppl. Table 5), indicating that methylation can affect gene regulation through various mechanisms, such as promoter accessibility or splice site manipulation. As illustrated in Figure 3B, DMEGs within the top 100 DMRs (ranked by absolute log_2_ FC) are predominantly found in promoter regions and show distinctly higher prevalence on chromosome 11.

To investigate which cell types might drive DNA methylation changes in BAT, we overlapped DMEGs from the *COLD*_*lean*_ condition with cell-type-specific marker genes derived from single-cell RNAseq (scRNAseq) of BAT from male mice exposed to cold (5 °C) versus thermoneutrality (30 °C) [42]. This analysis revealed that DMEGs were distributed across multiple cell types, with the highest proportions in vascular smooth muscle cells (VSM, 43 %), adipocytes (35 %), endothelial cells (EC, 31.8 %), and stromal populations, including *Pdgfra*^+^ adipocyte progenitor cells (APCs, 28.1 %) and pericytes (20.9 %). In contrast, immune cells contributed more modestly, with macrophages at 18.2 %, NK cells at 9.3 %, and T cell subsets (CD4^+^, CD8^+^, Treg) ranging from 5 to 9 %. Across nearly all cell types, most DMEGs belonged to the hypo up category - genes exhibiting hypomethylation accompanied by increased expression. This pattern was particularly pronounced in adipocytes (26.4 %) and vascular populations, including VSM (21.6 %) and EC (11.9 %). Hyper up regulated DMEGs were most notable in adipocytes (10 %), whereas hypo down regulated DMEGs were enriched in vascular and stromal populations, including VSM (15 %), EC (14.9 %), *Pdgfra*^*+*^ APCs (13.6 %), and to a lesser extent in adipocytes (2.8 %). Conversely, the fractions of hyper down DMEGs were relatively small, ranging from 1 to 6 % depending on the cell type (Figure 3C; Suppl. Table 5).

**Figure 4:**
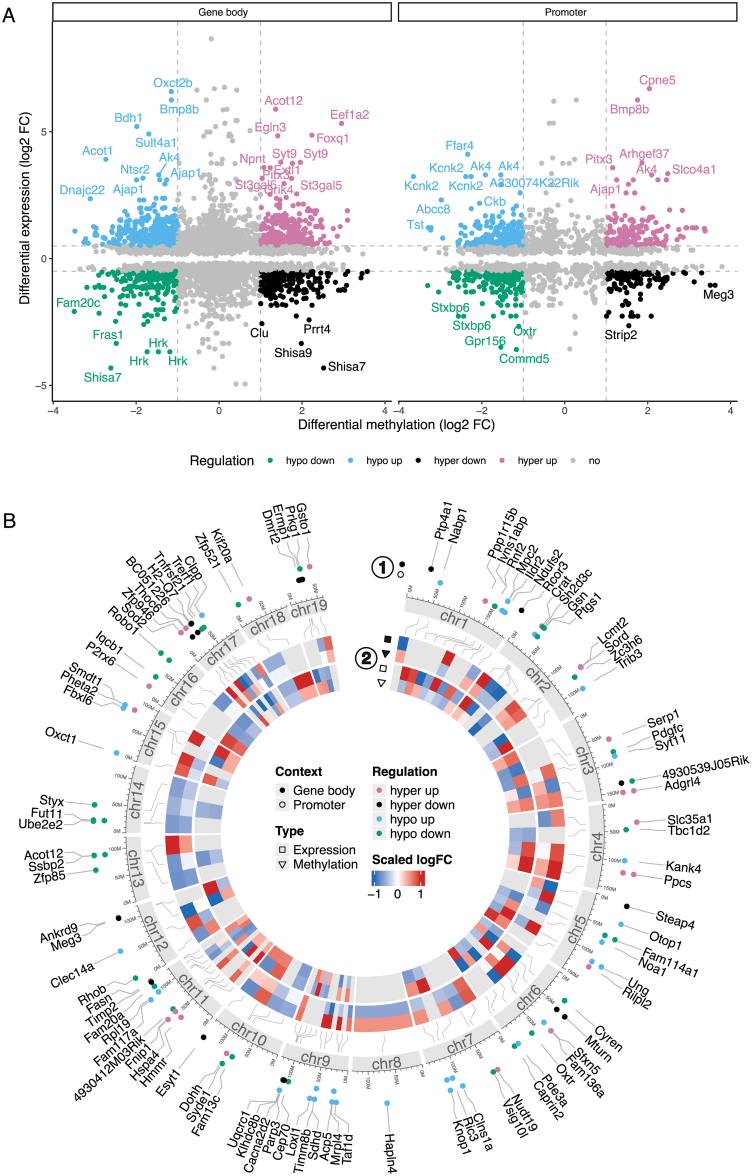
**Multi-layered insights into DMEGs under *COLD*_*DIO*_ condition.** (A) The scatter plot displays the log_2_ FC in gene expression and DNA methylation signals for all DMEGs in both gene body and promoter contexts under the *COLD*_*DIO*_ condition. Colors indicate the direction of regulation. DMEGs with absolute log_2_ FC > 0.5 in expression and >1 in methylation signal are highlighted, and labels are given for those DMEGs in the 5 % and 95 % quantile of expression and methylation log_2_ FC. (B) The circos visualization illustrates the DMEGs covering the top 100 most significant DMRs (ranked by log_2_ FC). This plot integrates multiple layers of information from outer to inner ring: Dot colors indicate the direction of regulation, while dot positions denote whether the DMR of the DMEG is located in the gene body or promoter context (1); a stylized ideogram of chromosomes provides a genomic reference frame; and a heatmap displays scaled log_2_ FC for both gene expression and DNA methylation signals in gene body and promoter contexts for each DMEG (2).

Within the *COLD*_*DIO*_ condition, we detected 1,001 DMEGs, corresponding to 19 % of all DEGs. These DMEGs corresponded to 1,886 unique DMR-intersecting DMPs, with 11 % of DMEGs associated with four or more DMPs (Suppl. Figure 6B). Similar to the *COLD*_*lean*_ condition, 60 % of DMEG-associated DMPs are located within gene bodies and 40 % in promoter regions (Figure 4A; Suppl. Table 6); however, the most highly regulated DMEGs do not exhibit enrichment in the promoter regions (Figure 4B).

Although we previously observed that the majority of significant DEGs overlapping between the *COLD*_*lean*_ and *COLD*_*DIO*_ conditions are regulated in a similar direction (Suppl. Figure 3A), we identified a contrasting pattern among DMEGs. The gene expression of DMEGs shows consistent regulatory patterns, with only 245 (*COLD*_*lean*_: 16 %; *COLD*_*DIO*_: 24 %) genes being upregulated and 159 (*COLD*_*lean*_: 10 %; *COLD*_*DIO*_: 16 %) genes being downregulated in both conditions (Suppl. Figure 3B; Suppl. Figure 7). In contrast, 73 % and 60 % are specifically regulated in the *COLD*_*lean*_ and *COLD*_*DIO*_ conditions, respectively, while only one gene (*guanylyl cyclase domain containing 1* (*Gucd1*)) is regulated in the opposite direction (Suppl. Figure 3B; Suppl. Figure 8). This suggests that the response to temperature conditions is strongly dependent on the specific environment and that methylation changes may be purposefully employed to adapt to different metabolic or physiological demands.

**Figure 5:**
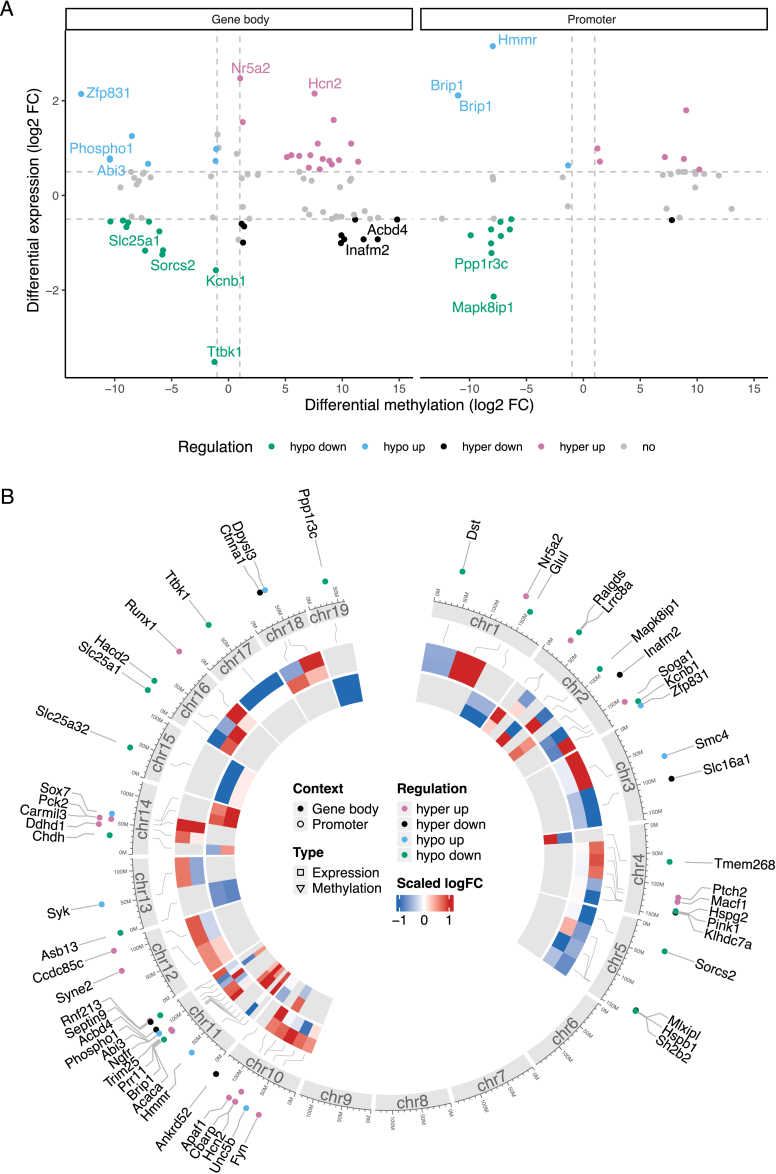
**Multi-layered insights into DMEGs under *ΔCOLD* condition.** (A) The scatter plot displays the log_2_ FC in gene expression and DNA methylation signals for all DMEGs in both gene body and promoter contexts under the Δ*COLD* condition. Colors indicate the direction of regulation. DMEGs with absolute log_2_ FC > 0.5 in expression and >1 in methylation signals are highlighted, and labels are given for those DMEGs in the 5 % and 95 % quantile of expression and methylation log_2_ FC. (B) The circos visualization illustrates all detected DMEGs in their respective DMRs. This plot integrates multiple layers of information from outer to inner ring: Dot colors indicate the direction of regulation, while dot positions denote whether the DMR of the DMEG is located in the gene body or promoter context (1); a stylized ideogram of chromosomes provides a genomic reference frame; and a heatmap displays scaled log_2_ FC for both gene expression and DNA methylation signals in gene body and promoter contexts for each DMEG (2).

Our previous findings revealed pronounced differences in the regulatory patterns of DMEGs between the *COLD*_*lean*_ and *COLD*_*DIO*_ conditions by emphasizing the direction of regulation. To further elucidate the nuances of metabolic state-specific regulation, we utilized the Δ*COLD* contrast to detect more subtle differences in regulatory effects including regulation strength, directly identifying metabolic state-related differences in DMEGs associated with BAT activation induced by cold exposure (Figure 5; Suppl. Table 7). For the Δ*COLD* contrast, we detected 56 DMEGs from only 4 % of all DEGs. A total of 65 distinct DMPs defined these DMEGs, and each DMEG is linked to at most three DMPs (Suppl. Figure 6C). Interestingly, the DMPs are primarily enriched in the gene bodies (69 %), suggesting that the regulation of these genes may be more influenced by post-transcriptional mechanisms in response to cold exposure.

### 3.4 Adaptive divergence in thermogenic signaling reflects metabolic stress in obese BAT

**Figure 6:**
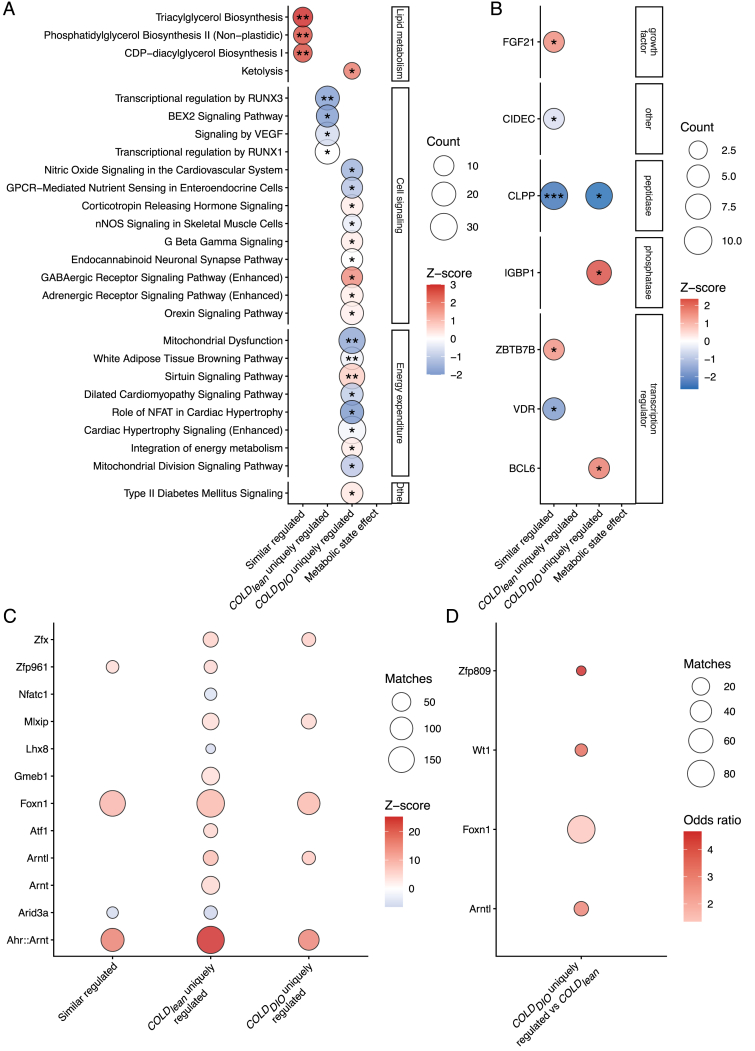
**Pathway and Transcription factor motif enrichment analysis.** (A, B) Ingenuity Pathway Analysis (IPA) with enrichments of DMEGs were categorized into four groups: those exhibiting similar regulation in both *COLD*_*lean*_ and *COLD*_*DIO*_ condition pairs, and those that are uniquely regulated in each individual condition (Δ*COLD, COLD*_*lean*_, and *COLD*_*DIO*_). Top ten statistically significant enrichments are shown for (A) canonical pathways and (B) upstream regulators. More than ten enrichments are listed in case of p-value ties. Circle sizes reflect the number of genes in each enrichment while the color highlights the activation Z-score. Significance levels for p after adjustment for multiple testing with Bonferroni-Holm are indicated as p < 0.05 (∗), p < 0.01 (∗∗). (C–D) Transcription factor binding site motif enrichments in genome sequences of + -20 nt windows up and downstream of locis from DMEG methylations. (C) DMEGs were categorized into three groups: those exhibiting similar regulation in both *COLD*_*lean*_ and *COLD*_*DIO*_ condition pairs, and those that are uniquely regulated in each individual condition (*COLD*_*lean*_, and *COLD*_*DIO*_). Enrichments were made in contrast to randomly sampled promoter sequences of the mm10 reference genome during an iterative process. All enrichments shown are statistically significant with FDR <0.001. (D) Motif enrichment of sequences from uniquely regulated DMEGs in *COLD*_*DIO*_ against all DMEGs in *COLD*_*lean*_ as physiological reference. One sided hypergeometric test was applied. Enrichments shown are filtered by p < 0.05.

For further characterization of the reported DMEGs, pathway enrichment analysis was performed using the IPA software. The DMEGs were categorized into four groups: those exhibiting similar regulation in both *COLD*_*lean*_ and *COLD*_*DIO*_ condition pairs, and those that are uniquely regulated in each individual condition (*COLD*_*lean*_, and *COLD*_*DIO*_) and Δ*COLD*. Querying the catalog for significant (BH-adjusted p < 0.05) canonical pathways (Figure 6A; Suppl. Table 8) shows similar activation of pathways with respect to lipid metabolism except for ketolysis which is only significantly activated in the *COLD*_*DIO*_ condition. Cell signaling and energy expenditure seem to be activated or inhibited more differentially between *COLD*_*lean*_ and *COLD*_*DIO*_ condition pairs. For example, the process of white adipose tissue browning is significantly inhibited in *COLD*_*DIO*_ but not in *COLD*_*lean*_. Additionally, type II diabetes mellitus signaling is significantly activated under *COLD*_*DIO*_ conditions but not under *COLD*_*lean*_. The upstream regulator enrichment analysis revealed that most regulators were involved in both metabolic states, similarly including growth factor FGF21 (fibroblast growth factor 21) as well as transcription regulators ZBTB7B (zinc finger and BTB domain containing 7 B), Vdr (vitamin D receptor), and Cidec (cell death inducing DFFA like effector C) (Figure 6B, Suppl. Table 9). While no significant activation is uniquely found in *COLD*_*lean*_ and Δ*COLD*, distinct regulatory effects are detected in *COLD*_*DIO*_ such as activation of phosphatase IGBP1 (immunoglobulin binding protein 1) and transcription regulator BCL6 (BCL6 transcription repressor).

### 3.5 Distinct transcription factor motifs are prevalent in DMEG sequences of all metabolic states

Transcription factor binding-site motive enrichment analysis identified nine significant TF-motifs in the set of similarly regulated DMEGs (overrepresented: N = 6, underrepresented: N = 3). In contrast, metabolic state-specific DMEGs showed much broader profiles: 28 TF-motifs (overrepresented: N = 12, underrepresented: N = 16) were uniquely regulated in *COLD*_*lean*_, and eight TF-motifs in *COLD*_*DIO*_ (overrepresented: N = 7, underrepresented: N = 1). The Δ*COLD* interaction set did show significant TF-motif enrichment (Suppl. Table 10).

Focusing on the strongest enrichments (FDR <0.001), the heterodimer AhrArnt (aryl-hydrocarbon receptoraryl hydrocarbon receptor nuclear translocator) and Foxn1 (forkhead box N1) emerged as the most strongly over-represented TF-motifs, exhibiting roughly a five and two-fold enrichment respectively relative to the background set (Figure 6C, Suppl. Table 10). Both appeared in the similarly regulated DMEG set as well as in the sets that were metabolic state-specific, indicating that their regulatory influence is largely independent of metabolic state and instead driven by the physiologic cold-induced regulatory architecture in BAT. When testing the effect of metabolic states, *COLD*_*DIO*_ reveals four overrepresented TF-motifs nominally enriched relative to *COLD*_*lean*_ (p < 0.05), including Arntl (basic helix-loop-helix ARNT like 1) and Foxn1, although none remained significant after FDR correction (Figure 6D, Suppl. Table 10). Thus, while subtle metabolic state-dependent differences may exist, the primary TF motif signature appears to be driven by cold exposure.

### 3.6 Metabolic states and cold exposure shape epigenetic regulation in BAT

**Figure 7:**
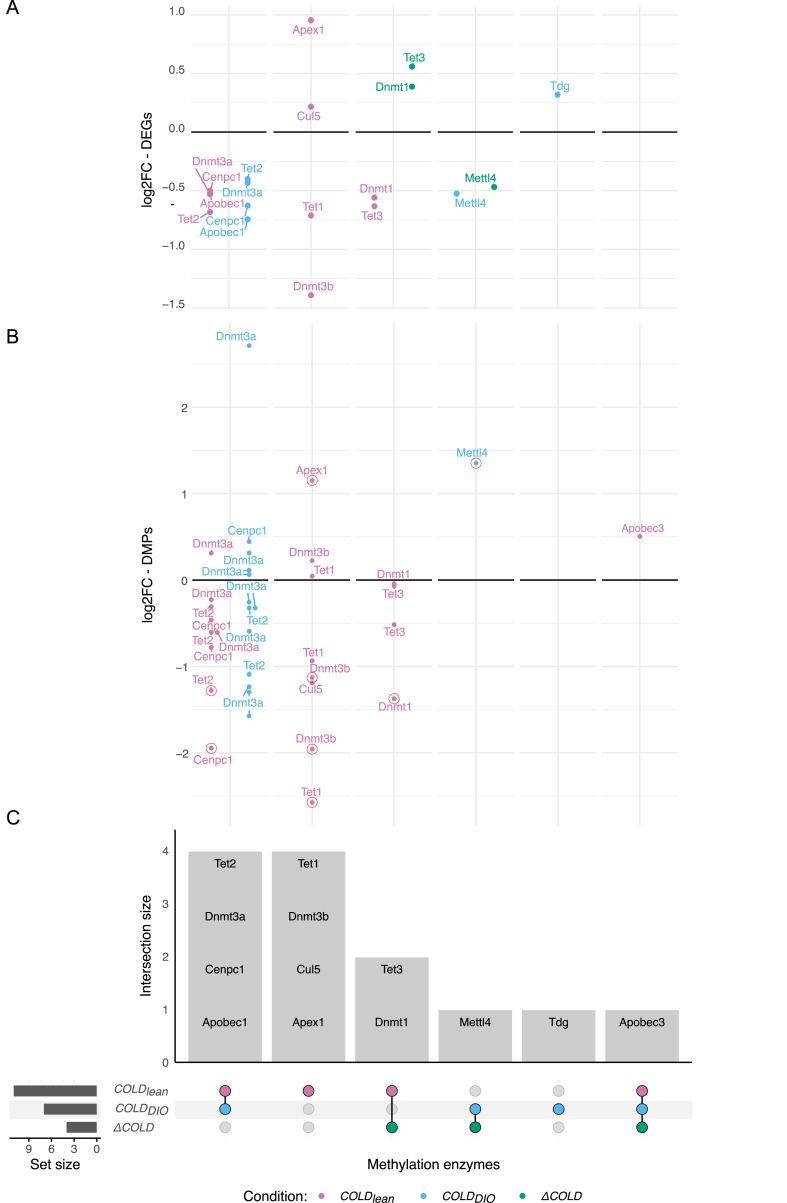
**Overview of methylation regulators in cold-induced BAT activation across metabolic conditions.** (A) Scatterplot showing the log_2_ fold-change of differentially expressed methylation regulators for each condition pair (*COLD*_*lean*_, *COLD*_*DIO*_, and Δ*COLD)* highlighted by color. (B) Scatterplot showing the log_2_ fold-change of DMPs within methylation regulator genes for each condition pair. DMPs meeting DMEG conditions are marked with red circles. (C) Upset plot illustrating the overlap of differentially expressed methylation regulators across the condition pairs.

To gain deeper insights into the epigenetic mechanisms that regulate BAT activation and thermogenesis under different metabolic conditions, we matched DEGs from each condition pair with the EpiFactors database (v2.0, https://epifactors.autosome.org, accessed on 10.09.2024), with the analysis being restricted to DNA-methylation-related factors, while histone-modifying enzymes, chromatin remodelers and RNA-based epigenetic regulators were omitted. As summarized in Figure 7A,C, we identified eleven differentially expressed methylation regulators for the *COLD*_*lean*_, seven for *COLD*_*DIO*_, and four for Δ*COLD* condition pairs. Notably, five regulators were found to be common to both the *COLD*_*lean*_ and *COLD*_*DIO*_ conditions including *Tet2* (*ten-eleven translocation methylase 2*), *Dnmt3* (*DNA methyltransferase 3*), *Cenpc1* (*centromere protein C*), *Apobec1* (*apolipoprotein B mRNA editing enzyme catalytic subunit 1*) which are all downregulated in *COLD*_*lean*_ *and COLD*_*DIO*_. This overlap indicates a conserved epigenetic mechanism that is essential for the physiological response to cold exposure, regardless of metabolic state. However, the majority of methylation enzymes exhibited unique regulation within each condition pair, highlighting the nuanced regulation of epigenetic mechanisms based on metabolic composition. For instance, *Tet3* and *Dnmt1* were differently regulated within the Δ*COLD* and *COLD*_*lean*_ conditions, while *Mettl4* (*methyltransferase-like protein 4*) and *Apobec3b* showed differential regulation within the Δ*COLD* contrast.

In addition to differential expression, we evaluated whether these methylation regulators also showed differential DNA methylation or coordinated methylation–expression changes, as illustrated in Figure 7B. Nearly all differentially expressed methylation regulators displayed differential methylation of at least one CpG site within a DMR. The only exceptions were *Apobec1* and *Tdg* (*thymine DNA glycosylase*), suggesting that their transcriptional changes occur independently of promoter or gene-body methylation. Several key regulators, including *Tet2*, *Cenpc1, Dnmt1*, and *Mettl4*, fulfilled DMEG criteria, reflecting coordinated alterations in both methylation and expression. This pattern suggests that cold exposure modulates components of the DNA-methylation machinery, with the specific regulatory changes differing across metabolic states.

## 4 Discussion

DNA methylation plays an important role in regulating the plasticity of BAT and contributes to the regulation and control of switching between thermoneutral and thermogenic states in response to environmental cues such as temperature and diet. Methylation changes in key thermogenic and metabolic genes, including *Ucp1*, *Prdm16*, and *Ppargc1a* govern both the differentiation of brown adipocyte precursors and BAT activity [7,8]. However, the extent to which DNA methylation directly drives cold-induced transcriptional changes in adult BAT remains incompletely understood, particularly under metabolically stressed conditions.

We aimed to explore how environmental factors and DNA methylation may influence BAT function. To investigate potential links between DNA methylation and gene expression, we integrated RNA-seq data with RRBS methylation profiles to identify genes with coordinated changes in expression and methylation (DMEGs) under different metabolic and thermal conditions. Rather than testing causality, this strategy was intended to capture condition-specific associations between epigenetic and transcriptional responses. This approach provides insights into BAT plasticity and adaptive thermogenesis, contributing to a foundational understanding that could inform future obesity-related research and interventions.

Consistent with BAT's physiological adaptation to cold, our data show that cold exposure robustly increases BAT activity regardless of metabolic state, evidenced by strong transcriptomic responses with 7,089 DEGs in lean and 5,249 DEGs in DIO mice. A substantial overlap (∼3,780 genes) between both metabolic state groups suggests a conserved “core” thermogenic program that remains inducible under obesogenic conditions. Notably, *Ucp1*, a central mediator of non-shivering thermogenesis, and *Letmd1* (*leucine zipper-EF-hand-containing transmembrane protein domain containing 1*), which is essential for mitochondrial integrity and cold response, were among the top upregulated genes across both metabolic states. Their consistent induction under cold exposure, aligns with previous reports of diet-insensitive thermogenic regulators [43,44]. Beyond this shared response, a distinct subset of genes exhibited metabolic-state-dependent regulation under cold exposure. Specifically, ∼1,360 genes were differentially expressed between the cold-exposed metabolic states (Δ*COLD*), with most downregulated in DIO mice, consistent with a high-fat diet impairing thermoregulatory capacity driven by mitochondrial dysfunction, inflammation, and insulin resistance [45,46]. This functional attenuation under high-fat diet conditions is also reflected among the most strongly regulated DEGs. Downregulation of *Hk2* (*hexokinase 2*), a key glycolytic enzyme, has been linked to reduced glucose uptake [47]. Similarly, reduced *Gpd1* (*glycerol-3-phosphate dehydrogenase 1*) expression, part of the glycerol-3-phosphate shuttle, may reflect compromised lipid metabolism. *Ucp1* stayed cold-inducible in all metabolic states but was lower in DIO mice, showing that thermogenic response is maintained but reduced under metabolic stress. This aligns with reports of reduced BAT activity in obesity despite retained gene expression [43]. Together, these findings suggest that obesity dampens but does not abolish the transcriptional capacity of BAT to respond to cold. Our results of *COLD*_*lean*_ are in line with those of Taylor et al. (2024) [36], who performed a comparable study in lean chow-fed mice exposed to severe cold (8 °C) versus thermoneutrality (28 °C). The strong overlap of DEGs highlights the reproducibility of cold-induced BAT transcriptional responses across independent studies and supports the generalizability of our findings.

To identify epigenetically regulated genes, we correlated methylation changes (M-values of DMPs overlapping DMRs) with gene expression levels (DEGs) to define DMEGs. While these correlations suggest potential regulatory links, they do not demonstrate that methylation changes precede or directly cause transcriptional regulation, as gene expression is also influenced by other epigenetic modifications and transcription factors [36,48,49]. Accordingly, our analysis should be interpreted as identifying coordinated patterns rather than mechanistic drivers. More comprehensive approaches integrating histone modifications, proteomics, or single-cell epigenomics could further elucidate the complex regulation within heterogeneous BAT cell populations [50]. Compared to generalized linear models with distributional assumptions [51,52], our site-specific correlation approach offers higher sensitivity for detecting local methylation–expression relationships, though it may miss broader regulatory patterns and is more sensitive to noise. Our thresholds for defining significant DMEGs were set at |log FC| >0.5 for expression changes and |log FC| >1 for methylation differences; correlations were considered significant if |r| >0.5 and p < 0.05. These analytical choices balance sensitivity and specificity but also limit detection power given our sample size. When DMEGs overlapped promoter and gene body regions, assignment was based on the strongest significance, which enhances interpretability but may introduce bias due to regional variability or technical factors.

By analyzing DMEGs, ∼3,100 DMEGs (21 % of DEGs) were identified in *COLD*_*lean*_, with a roughly equal distribution between promoter and gene body methylation. In contrast, DIO mice exhibited only ∼1,900 DMEGs (19 % of DEGs), with a higher proportion of gene body methylation. As gene body methylation is often linked to transcript fine-tuning rather than activation, this shift may reflect altered transcriptional modulation rather than a complete loss of epigenetic regulation under obesogenic conditions [53]. Importantly, despite substantial overlap at the DEG level, DMEGs were largely condition-specific. This indicates that the metabolic state influences which methylation–expression relationships are engaged during cold exposure, rather than enforcing a uniform epigenetic response. In this context, DNA methylation may shape the direction and magnitude of transcriptional adaptation rather than serving as a binary regulatory switch.

To gain further insight into the cellular context of these DNA methylation changes, we mapped DMEGs of the *COLD*_*lean*_ condition onto cell-type-specific markers derived from male mice scRNAseq data of cold-exposed BAT [42]. This analysis revealed that the majority of DMEGs belonged to the hypo up category, particularly in adipocytes, vascular cells, and *Pdgfra*^*+*^ adipocyte progenitors, suggesting coordinated activation of thermogenic programs and vascular remodeling. Hypo down DMEGs were enriched in vascular and stromal populations, indicating selective repression of pathways supporting tissue structure and function. Hyper up DMEGs were primarily observed in adipocytes, whereas hyper down DMEGs were rare (1–6 %), suggesting that methylation-associated gene repression is not a dominant feature of the cold response. These distributions are consistent with a model of selective epigenetic engagement across BAT cell populations. Furthermore, the observed cell type-specific DMEG distributions are consistent with prior evidence of increased intercellular signaling among adipocytes, vascular, stromal, and immune cells under cold conditions in lean chow-fed mice, although direct functional roles of methylation in these interactions remain to be determined [42].

Pathway enrichment analysis further supports this adaptive divergence of DMEGs and highlighting the metabolic state-dependent rewiring of thermogenic signaling. Cold exposure activated distinct lipid metabolic pathways (triacylglycerol, phosphatidylglycerol, and CDP−diacylglycerol biosynthesis) in both metabolic groups, however, ketolysis was selectively enriched in DIO mice. In line with previous findings that HFD suppresses ketone body utilization for lipogenesis in BAT via downregulation of *acetoacetyl-CoA synthetase* (*Aacs*) [54], our differential gene expression analysis shows that *Aacs* was significantly upregulated by cold in both metabolic groups, but this response was markedly stronger in lean mice (log_2_ FC: 1.77) than in DIO mice (log_2_ FC: 0.74), with a negative interaction effect (Δ*COLD* log_2_ FC: 0.81). These findings indicate a quantitative attenuation rather than a complete loss of ketone body recycling under DIO conditions, potentially limiting metabolic flexibility and compensatory lipid synthesis in insulin-resistant BAT. Consistently, only cold-exposed DIO mice showed activation of the Type II Diabetes Mellitus signaling pathway under cold stress, reflecting a diabetogenic transcriptional environment marked by insulin resistance and impaired glucose homeostasis [55]. Together, these results suggest that cold exposure incompletely counteracts metabolic dysfunction in obese BAT.

Further, cold-exposed DIO mice uniquely activated Sirtuin signaling, which may represent a compensatory response to metabolic or mitochondrial stress, while suppressing pathways related to mitochondrial dysfunction and white adipose tissue browning, indicating a shift from efficient thermogenesis to cellular maintenance [56]. Selective activation of neuroendocrine pathways (e.g., corticotropin-releasing hormone and orexin signaling) may similarly reflect rewiring of central and peripheral regulatory mechanisms to compensate for reduced intrinsic thermogenic capacity [57]. Suppression of nitric oxide and neuronal nitric oxide synthase (nNOS) signaling suggests impaired vascular adaptation, although functional consequences were not directly assessed in this study.

We identified several epigenetic regulators differentially expressed in response to cold in a metabolic-state–dependent manner. Core factors such as *Tet2*, *Dnmt3*, *Cenpc1*, and *Apobec1* responded similarly under both metabolic conditions, suggesting a shared epigenetic response associated with cold exposure. In contrast, regulators such as *Mettl4*, *Tet3*, and *Dnmt1* showed metabolic-state–specific regulation. While these patterns are consistent with altered epigenetic flexibility in obesity [[58], [59], [60], [61]], their direct functional contribution to BAT thermogenesis remains speculative. TF motif enrichment highlighted AhrArnt, key regulators of lipid metabolism and energy homeostasis in adipocytes [62], suggesting potential regulatory involvement rather than confirming functional upstream control.

By conducting *in vitro* methylation analyses, we confirmed that altering methylation levels affects expression of selected BAT genes such as *Bmp8b* and *Cpne5*. However, these experiments do not exclude contributions from other epigenetic mechanisms, particularly given the broad effects of methylation-modifying agents. Taken together, the data support an association between DNA methylation dynamics and BAT gene regulation, while underscoring the need for targeted functional studies.

### 4.1 Limitations

A limitation arises from age differences between diet groups (11 weeks chow versus 22 weeks HFD). First, the difference in bodyweight between chow and HFD mice may have partially arisen from the age gap, as there is still a slow increase in body weight beyond adulthood at 12 weeks of age [41]. However, male chow fed mice at 22 weeks of age would reach approximately 30 g bodyweight which still is significantly different from mice on HFD at the same age. Second, although BAT function declines with age, mice used in this study are of a comparable age-range related to BAT function. Studies show that mice aged between 12 weeks and six months present a stable metabolic active BAT, with stable mitochondrial content and thermogenic potential before these factors start to decline and lipid accumulation increases [[38], [39], [40]]. Although we cannot bioinformatically address this issue in our comparative analyses, we believe that age is not a main driving factor of the here observed effects as we addressed a comparable age range regarding bodyweight and cold adaptation of BAT. Anyway, regardless of BAT function, global DNA methylation reduces with age while specifically promoter regions tend to get hypermethylated with age [63]. While we cannot address the age factor in our analyses due to group specific age differences, we expect a limited impact of age on our results based on comparing mice in a suitable age range meaning only “young adult mice” [64].

A limitation of the late-onset HFD exposure model used in this study is that it does not reflect the complex interplay between intergenerational and early developmental epigenetic programming due to HFD exposure [65]. However, late-exposure models offer key benefits. By introducing a high-fat diet after the early developmental phase, these models help isolate epigenetic changes arising from direct dietary impact rather than developmental programming. In combination with other exposure models, this distinction enables the assessment of which molecular pathways remain plastic and which have been permanently established by early exposure [66]. Furthermore, when combined with information on parental or early-life programming, late-exposure paradigms may help determine whether inherited epigenetic marks sensitize or protect against later dietary challenges. This would be an important step toward understanding the cumulative or synergistic effects across generations.

A further limitation is the use of only male mice, which may limit generalizability to females. However, given the known sex-specific differences in adipose biology, BAT activity, and epigenetic regulation, addressing only males is beneficial regarding the addressed methodology. Future studies should include female mice to assess potential sex-by-diet or sex-by-temperature interactions. Small sample sizes (7–8 per group) limit statistical power to detect subtle effects.

In addition, RRBS data provides only partial genome coverage, primarily focusing on CpG-rich regions, potentially overlooking important regulatory areas outside these regions. In contrast, whole-genome bisulfite sequencing could provide more comprehensive methylation profiling. Furthermore, this study focused only on methylation changes associated with differentially expressed genes aiming to add knowledge on DNA-methylation regulated gene transcription. However, with this approach we did not reflect the occurrence of methylation changes across all genes limiting insights about overall methylation dynamics under the tested conditions.

## 5 Conclusions

In conclusion, this study provides the evidence that cold-induced DNA methylation remodeling in BAT is influenced by metabolic context, particularly under obesogenic conditions. While cold exposure activates a conserved thermogenic transcriptional program, obesogenic conditions are associated with altered methylation–expression relationships that may constrain full BAT activation. Our findings support a model in which DNA methylation acts as a modulatory layer rather than a primary driver of cold-induced transcription, with its contribution shaped by metabolic state. By identifying both shared and context-specific methylation-associated regulators, this work provides a framework for future mechanistic studies aimed at defining how epigenetic regulation contributes to BAT plasticity in metabolic disease.

## CRediT authorship contribution statement

**Tobias Hagemann:** Writing – review & editing, Writing – original draft, Visualization, Methodology, Formal analysis, Data curation, Conceptualization. **Anne Hoffmann:** Writing – review & editing, Writing – original draft, Supervision, Methodology, Investigation, Formal analysis, Data curation, Conceptualization. **Kerstin Rohde-Zimmermann:** Writing – review & editing, Writing – original draft, Validation, Methodology, Investigation, Formal analysis, Data curation, Conceptualization. **Helen Broghammer:** Writing – review & editing, Validation, Investigation. **Lucas Massier:** Writing – review & editing. **Peter Kovacs:** Writing – review & editing. **Michael Stumvoll:** Writing – review & editing. **Matthias Blüher:** Writing – review & editing, Funding acquisition. **John T. Heiker:** Writing – review & editing, Writing – original draft, Supervision, Project administration, Funding acquisition, Conceptualization. **Juliane Weiner:** Writing – review & editing, Writing – original draft, Validation, Investigation, Funding acquisition, Conceptualization.

## Funding

This work was funded or supported by grants of the 10.13039/501100001659Deutsche Forschungsgemeinschaft, project number 209933838 (SFB1052 “Obesity Mechanisms”: B1 to MB, C7 to JTH) and by the Formel1 Nachwuchsförderung of the Medical Faculty of the University of Leipzig (JW). HB is supported by a doctoral scholarship of the Studienstiftung des Deutschen Volkes. LM is supported by the Swedish Research Council, the European Association for the Study of Diabetes and the German Diabetes Association.

## Declaration of competing interest

The authors declare the following financial interests/personal relationships which may be considered as potential competing interests: MB received honoraria as a consultant and speaker from Amgen, AstraZeneca, Bayer, Boehringer-Ingelheim, Lilly, Novo Nordisk, Novartis, and Sanofi. All other authors declare no conflicts of interest. The funders had no role in the design of the study, the collection, analyses, or interpretation of data, in the decision to publish the results and the writing of the manuscript.

## Data Availability

Raw RNAseq and RRBS data have been deposited in the Sequence Read Archive (SRA, https://www.ncbi.nlm.nih.gov/sra/) under the BioProject number PRJNA1294891.

## References

[1] Kusminski C.M., Bickel P.E., Scherer P.E. (2016). Targeting adipose tissue in the treatment of obesity-associated diabetes. Nat Rev Drug Discov.

[2] Saito M., Matsushita M., Yoneshiro T., Okamatsu-Ogura Y. (2020). Brown adipose tissue, diet-induced thermogenesis, and thermogenic food ingredients: from mice to men. Front Endocrinol.

[3] U Din M., Saari T., Raiko J., Kudomi N., Maurer S.F., Lahesmaa M. (2018). Postprandial oxidative metabolism of human brown fat indicates thermogenesis. Cell Metab.

[4] Chouchani E.T., Kazak L., Spiegelman B.M. (2019). New advances in adaptive thermogenesis: UCP1 and beyond. Cell Metab.

[5] Rosenbaum M., Leibel R.L. (2010). Adaptive thermogenesis in humans. Int J Obes.

[6] Scheele C., Wolfrum C. (2020). Brown adipose crosstalk in tissue plasticity and human metabolism. Endocr Rev.

[7] Xiao H., Kang S. (2019). The role of DNA methylation in thermogenic adipose biology. Epigenetics.

[8] Wang S., Cao Q., Cui X., Jing J., Li F., Shi H. (2021). Dnmt3b deficiency in Myf5+-Brown fat precursor cells promotes obesity in female mice. Biomolecules.

[9] Teperino R. (2025). Intergenerational metabolic effects of cold exposure. Nat Metab.

[10] Sun S., Hood M., Scott L., Peng Q., Mukherjee S., Tung J. (2017). Differential expression analysis for RNAseq using poisson mixed models. Nucleic Acids Res.

[11] Skinner M.K. (2018). Preconception cold–induced epigenetic inheritance. Nat Med.

[12] Zhang Q., Xiao X., Zheng J., Li M., Yu M., Ping F. (2021). Maternal high-fat diet disturbs the DNA methylation profile in the brown adipose tissue of offspring mice. Front Endocrinol.

[13] Madden C.J., Morrison S.F. (2016). A high-fat diet impairs cooling-evoked brown adipose tissue activation via a vagal afferent mechanism. Am J Physiol Endocrinol Metab.

[14] Bolger A.M., Lohse M., Usadel B. (2014). Trimmomatic: a flexible trimmer for Illumina sequence data. Bioinforma Oxf Engl.

[15] Dobin A., Davis C.A., Schlesinger F., Drenkow J., Zaleski C., Jha S. (2013). STAR: ultrafast universal RNA-seq aligner. Bioinforma Oxf Engl.

[16] Liao Y., Smyth G.K., Shi W. (2014). featureCounts: an efficient general purpose program for assigning sequence reads to genomic features. Bioinforma Oxf Engl.

[17] Love M.I., Huber W., Anders S. (2014). Moderated estimation of fold change and dispersion for RNA-seq data with DESeq2. Genome Biol.

[18] Di Tommaso P., Chatzou M., Floden E.W., Barja P.P., Palumbo E., Notredame C. (2017). Nextflow enables reproducible computational workflows. Nat Biotechnol.

[19] Ewels P., Hammarén R., Peltzer A., phue, Sven F., Tommaso P.D. (2019). Nf-Core/methylseq..

[20] Martin M. (2011). Cutadapt removes adapter sequences from high-throughput sequencing reads. EMBnetJournal.

[21] Krueger F., Andrews S.R. (2011). Bismark: a flexible aligner and methylation caller for bisulfite-seq applications. Bioinformatics.

[22] Ritchie M.E., Phipson B., Wu D., Hu Y., Law C.W., Shi W. (2015). Limma powers differential expression analyses for RNA-Sequencing and microarray studies. Nucleic Acids Res.

[23] Müller F., Scherer M., Assenov Y., Lutsik P., Walter J., Lengauer T. (2019). RnBeads 2.0: comprehensive analysis of DNA methylation data. Genome Biol.

[24] Durinck S., Moreau Y., Kasprzyk A., Davis S., De Moor B., Brazma A. (2005). BioMart and bioconductor: a powerful link between biological databases and microarray data analysis. Bioinformatics.

[25] Haider S., Waggott D., Boutros P.C. bedr: genomic region processing using tools such as ’BEDTools’, “BEDOPS”and “Tabix”. 2025. https://github.com/uclahs-cds/package-bedr.

[26] Lawrence M., Huber W., Pagès H., Aboyoun P., Carlson M., Gentleman R. (2013). Software for computing and annotating genomic ranges. PLoS Comput Biol.

[27] Herve M. RVAideMemoire: Testing and Plotting Procedures for Biostatistics. 2023. 10.32614/CRAN.package.RVAideMemoire

[28] Microsoft, Weston S. foreach. foreach: Provides Foreach Looping Construct. 2022. https://github.com/revolutionanalytics/foreach.

[29] Krämer A., Green J., Pollard J., Tugendreich S. (2014). Causal analysis approaches in ingenuity pathway analysis. Bioinformatics.

[30] Pederson S. (2025). motifTestR: perform key tests for binding motifs in sequence data. ..

[31] The Bioconductor Dev Team. BSgenome.Mmusculus.UCSC.mm10.masked: Full masked genome sequences for Mus musculus (UCSC genome mm10, based on GRCm38.p6). 2021. 10.18129/B9.bioc.BSgenome.Mmusculus.UCSC.mm10.masked.

[32] Rauluseviciute I., Riudavets-Puig R., Blanc-Mathieu R., Castro-Mondragon J.A., Ferenc K., Kumar V. (2024). Jaspar 2024: 20th anniversary of the open-access database of transcription factor binding profiles. Nucleic Acids Res.

[33] Paul Shannon M.R. (2017). MotifDb.

[34] Klein J., Fasshauer M., Klein H.H., Benito M., Kahn C.R. (2002). Novel adipocyte lines from brown fat: a model system for the study of differentiation, energy metabolism, and insulin action. Bioessays.

[35] Weiner J., Rohde K., Krause K., Zieger K., Klöting N., Kralisch S. (2017). Brown adipose tissue (BAT) specific vaspin expression is increased after obesogenic diets and cold exposure and linked to acute changes in DNA-methylation. Mol Metabol.

[36] Taylor B.C., Steinthal L.H., Dias M., Yalamanchili H.K., Ochsner S.A., Zapata G.E. (2024). Histone proteoform analysis reveals epigenetic changes in adult mouse brown adipose tissue in response to cold stress. Epigenetics Chromatin.

[37] Nedergaard J., Cannon B. (2014). The browning of white adipose tissue: some burning issues. Cell Metab.

[38] Cannon B., De Jong J.M.A., Fischer A.W., Nedergaard J., Petrovic N. (2020). Human brown adipose tissue: classical brown rather than brite/beige?. Exp Physiol.

[39] Silva G.D.N., Amato A.A. (2022). Thermogenic adipose tissue aging: mechanisms and implications. Front Cell Dev Biol.

[40] Zoico E., Rubele S., De Caro A., Nori N., Mazzali G., Fantin F. (2019). Brown and beige adipose tissue and aging. Front Endocrinol.

[41] The Jackson Laboratory (2025). Body Weight Information for B6J (Stock Number 000664)..

[42] Shamsi F., Zheng R., Ho L.-L., Chen K., Tseng Y.-H. (2023). Comprehensive analysis of intercellular communication in the thermogenic adipose niche. Commun Biol.

[43] Fromme T., Klingenspor M. (2011). Uncoupling protein 1 expression and high-fat diets. Am J Physiol Regul Integr Comp Physiol.

[44] Choi K.-M., Kim J.H., Kong X., Isik M., Zhang J., Lim H.-W. (2021). Defective brown adipose tissue thermogenesis and impaired glucose metabolism in mice lacking Letmd1. Cell Rep.

[45] Bournat J.C., Brown C.W. (2010). Mitochondrial dysfunction in obesity. Curr Opin Endocrinol Diabetes Obes.

[46] Maliszewska K., Kretowski A. (2021). Brown adipose tissue and its role in insulin and glucose homeostasis. Int J Mol Sci.

[47] Shimobayashi M., Thomas A., Shetty S., Frei I.C., Wölnerhanssen B.K., Weissenberger D. (2023). Diet-induced loss of adipose hexokinase 2 correlates with hyperglycemia. eLife.

[48] Kouzarides T. (2007). Chromatin modifications and their function. Cell.

[49] Lee T.I., Young R.A. (2013). Transcriptional regulation and its misregulation in disease. Cell.

[50] Smallwood S.A., Lee H.J., Angermueller C., Krueger F., Saadeh H., Peat J. (2014). Single-cell genome-wide bisulfite sequencing for assessing epigenetic heterogeneity. Nat Methods.

[51] Gevaert O., Tibshirani R., Plevritis S.K. (2015). Pancancer analysis of DNA methylation-driven genes using MethylMix. Genome Biol.

[52] Mishra N.K., Guda C. (2017). Genome-wide DNA methylation analysis reveals molecular subtypes of pancreatic cancer. Oncotarget.

[53] Moore L.D., Le T., Fan G. (2013). DNA methylation and its basic function. Neuropsychopharmacology.

[54] Yamasaki M., Hasegawa S., Ozaki S., Imai M., Saito D., Takahashi N. (2023). High-fat-diet suppressed ketone body utilization for lipogenic pathway in brown adipose tissues. Metabolites.

[55] Chondronikola M., Volpi E., Børsheim E., Porter C., Saraf M.K., Annamalai P. (2016). Brown adipose tissue activation is linked to distinct systemic effects on lipid metabolism in humans. Cell Metab.

[56] Escalona-Garrido C., Vázquez P., Mera P., Zagmutt S., García-Casarrubios E., Montero-Pedrazuela A. (2020). Moderate SIRT1 overexpression protects against brown adipose tissue inflammation. Mol Metabol.

[57] Madden C.J., Tupone D., Morrison S.F. (2012). Orexin modulates brown adipose tissue thermogenesis. Biomol Concepts.

[58] Zhang Z., Hou Y., Wang Y., Gao T., Ma Z., Yang Y. (2020). Regulation of adipocyte differentiation by METTL4, a 6 mA methylase. Sci Rep.

[59] Jung B.C., You D., Lee I., Li D., Schill R.L., Ma K. (2023). TET3 plays a critical role in white adipose development and diet-induced remodeling. Cell Rep.

[60] Park J., Lee D.H., Ham S., Oh J., Noh J.-R., Lee Y.K. (2022). Targeted erasure of DNA methylation by TET3 drives adipogenic reprogramming and differentiation. Nat Metab.

[61] Kajimura S. (2021). The epigenetic regulation of adipose tissue plasticity. Proc Natl Acad Sci.

[62] Li X., Wang S., Mao X., Fang M., Liu X., Jiang J. (2025). AhR in biological processes of adipocytes and lipid metabolism in obesity: friend and foe. Life Sci.

[63] Perez-Correa J.-F., Tharmapalan V., Geiger H., Wagner W. (2022). Epigenetic clocks for mice based on age-associated regions that are conserved between mouse strains and human. Front Cell Dev Biol.

[64] Berry D.C., Stenesen D., Zeve D., Graff J.M. (2013). The developmental origins of adipose tissue. Development.

[65] King S.E., Skinner M.K. (2020). Epigenetic transgenerational inheritance of obesity susceptibility. Trends Endocrinol Metabol.

[66] Hinte L.C., Castellano-Castillo D., Ghosh A., Melrose K., Gasser E., Noé F. (2024). Adipose tissue retains an epigenetic memory of obesity after weight loss. Nature.

