# Supplementary material for: Diet and temperature interactively impact brown adipose tissue gene regulation controlled by DNA methylation
Source: Mol Metab. 2026 Jan 1;104:102315. doi: 10.1016/j.molmet.2025.102315 (PMC12828542; doi:10.1016/j.molmet.2025.102315)
Supplement: Multimedia component 2 [file mmc2.pdf]

Supplementary Information

Diet and temperature interactively impact brown adipose tissue gene regulation controlled by DNA methylation

Tobias Hagemann, Anne Hoffmann, Kerstin Rohde-Zimmermann, Helen Broghammer, Lucas Massier, Peter Kovacs, Michael Stumvoll, Matthias Blüher, John T. Heiker and Juliane Weiner

Supplementary Figure 1

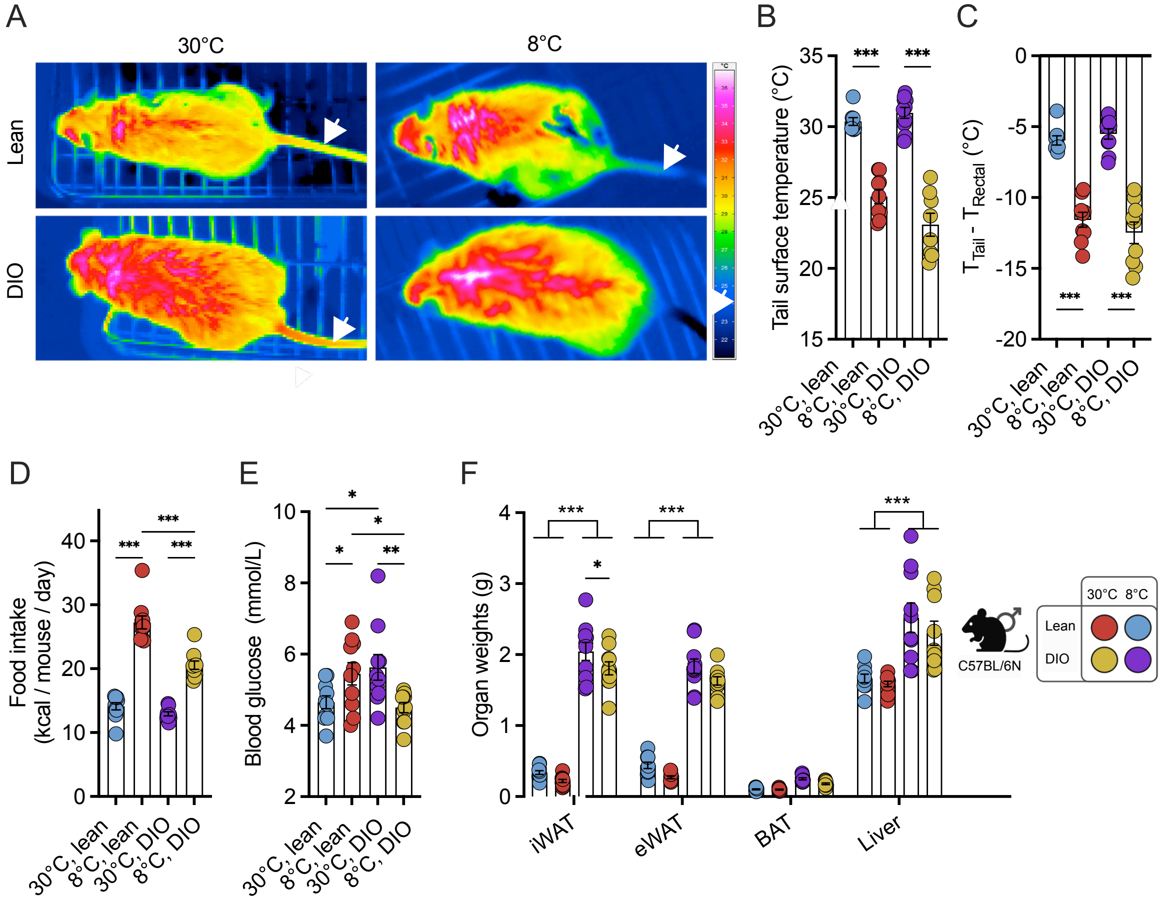

Supplementary Figure 1: Extended phenotyping of lean and diet-induced obese mice housed at 30°C or 8°C. Overview thermal images from brown adipose tissue (BAT) (A), tail surface temperature (B) and heat loss (Ttail – Trectal; (C) in lean (chow-fed) and diet-induced obese (DIO; high-fat diet (HFD)-fed) mice housed at thermoneutrality (30°C) or in the cold (8°C) for 7 days. Average daily calory intake (D), final fasted blood glucose (E) and absolute organ weights (F) of inguinal white adipose tissue (iWAT), epididymal white adipose tissue (eWAT), BAT and liver of lean and DIO mice housed at thermoneutrality (30°C) or in the cold (8°C) for 7 days. N = 7-8 per condition. Statistical significance was evaluated one-way ANOVA with Tukey's post-hoc test (B-D) or uncorrected Fischer's LDS (E) or two-way ANOVA with uncorrected Fischer's LDS (F). p <0.05 (*), p <0.01 (**), p <0.001 (***).

Supplementary Figure 2

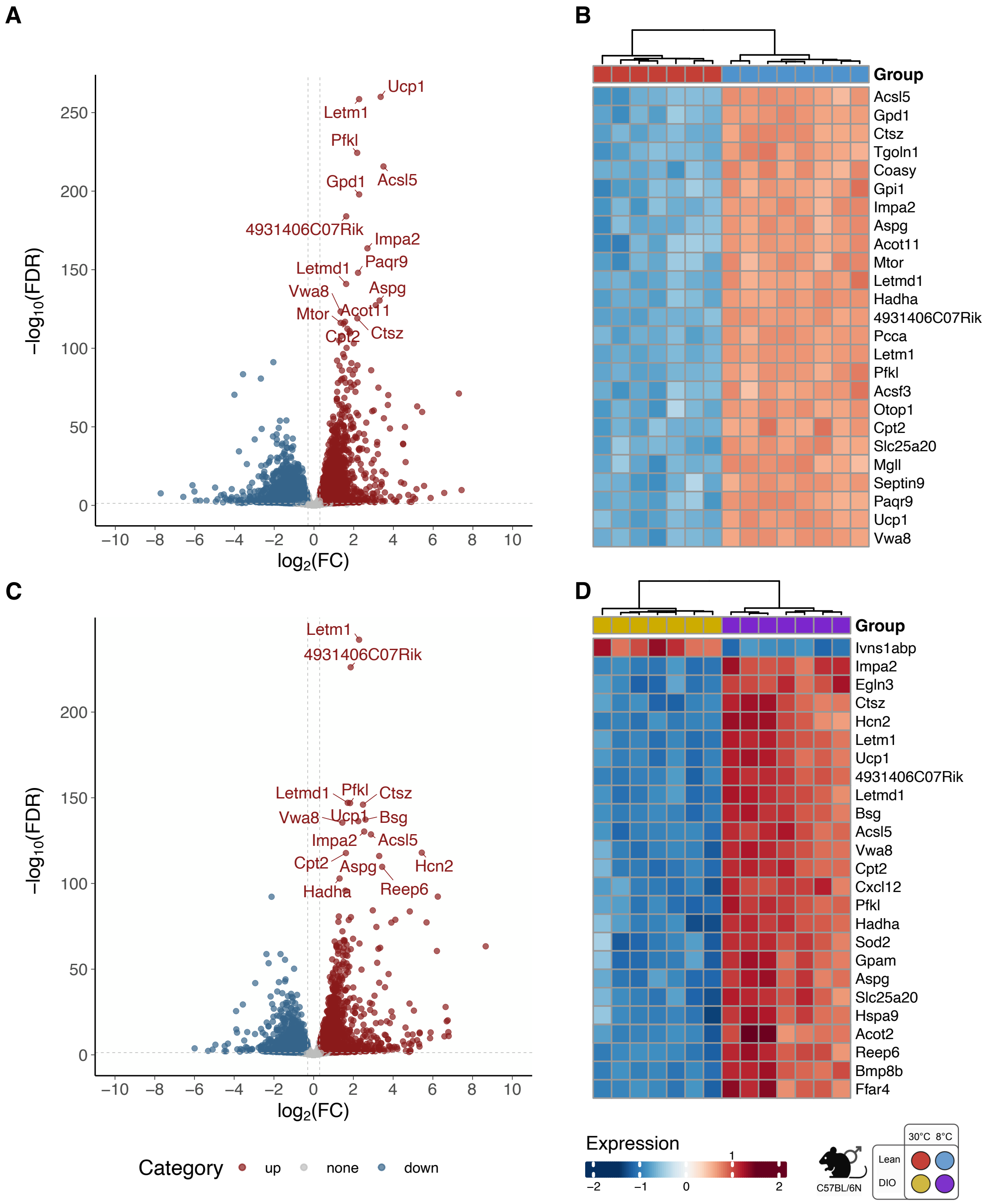

Supplementary Figure 2: Differential gene expression in response to cold exposure in lean and DIO mice. Summary of differential gene expression signals for cold-induced BAT activation in (A, B) lean normal-weight control mice ($COLD_{lean}$) and (C, D) DIO mice ($COLD_{DIO}$). Volcano plots (A, C) and heatmaps (B, D) display differentially expressed genes (DEGs) based on $\log_2$ fold change (FC) and false discovery rate (FDR) signals and show clustering of the top 25 DEGs, respectively.

Supplementary Figure 3

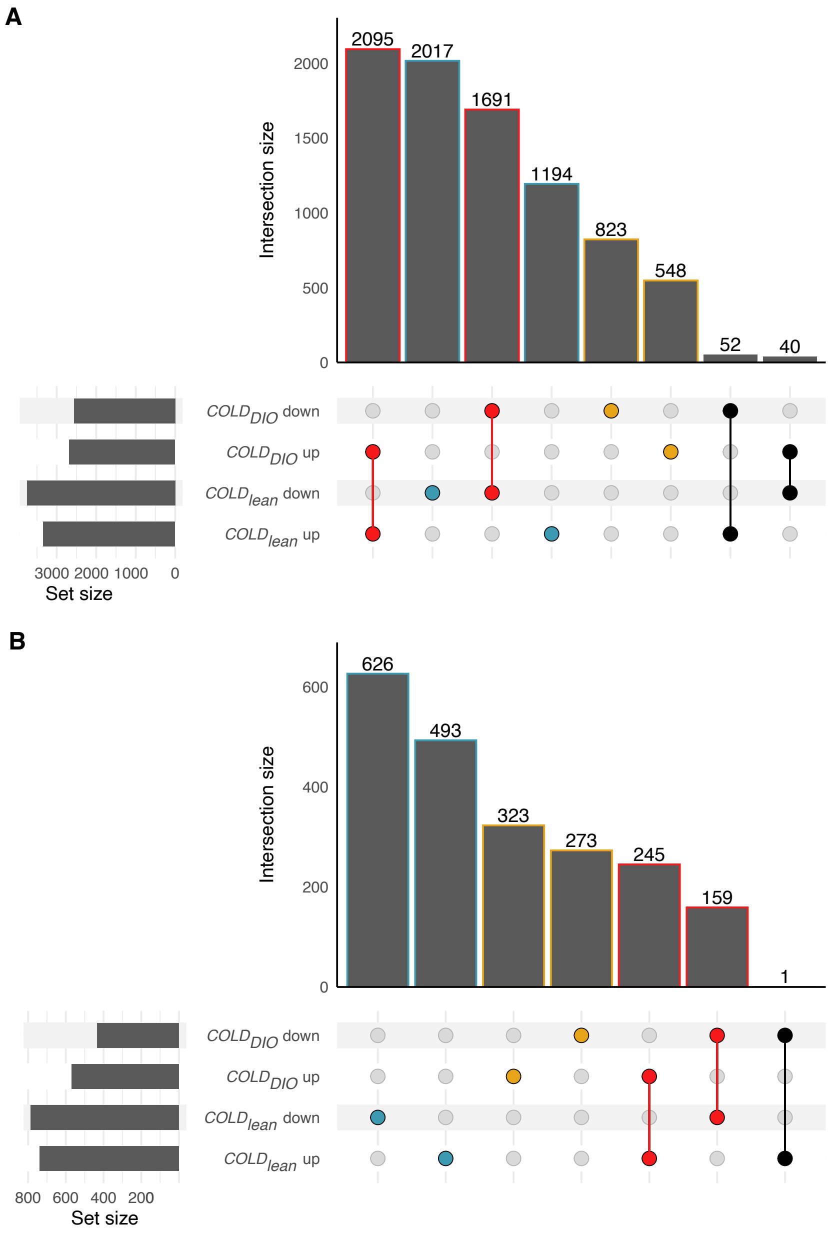

Supplementary Figure 3: Overlap of DEGs and DMEGs between the $COLD_{lean}$ and $COLD_{DIO}$ conditions. Upset plots display the overlap of (A) DEGs and (B) differentially methylated and expressed genes (DMEGs) for the $COLD_{lean}$ and $COLD_{DIO}$ condition pairs, categorized by positive (up) and negative (down) regulation. Significant DEGs or DMEGs that show the same and contrary regulatory direction in both conditions are highlighted in red and black respectively. Those significant only in the $COLD_{lean}$ condition are shown in blue, while those significant only in the $COLD_{DIO}$ condition are marked in yellow. Genes are counted uniquely, even though multiple DMEGs may be associated with the same gene. Set size indicates the total number of DEGs or DMEGs.

Supplementary Figure 4

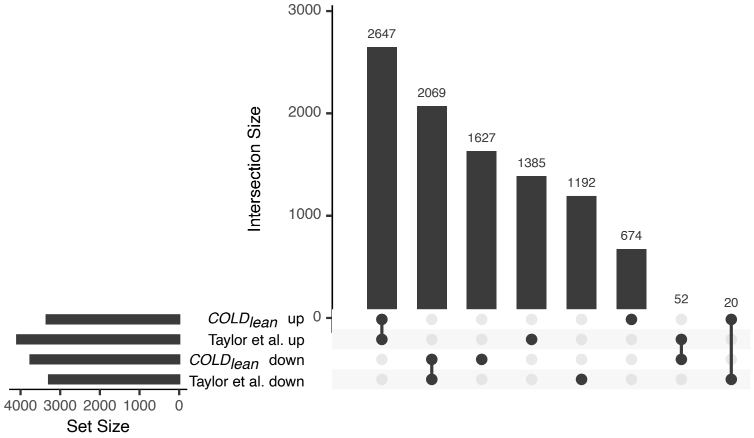

Supplementary Figure 4: Overlap of DEGs in the *COLD$_{lean}$* condition with published data. Upset plots show the intersection of DEGs from the *COLD$_{lean}$* condition and DEGs reported by Tayler et al. (2024) [1], for chow-fed lean male mice (8°C versus 28°C), categorized by positive (up) and negative (down) regulation. DEGs with adj. P >0.05 were compared. Set size indicates the total number of DEGs.

Supplementary Figure 5

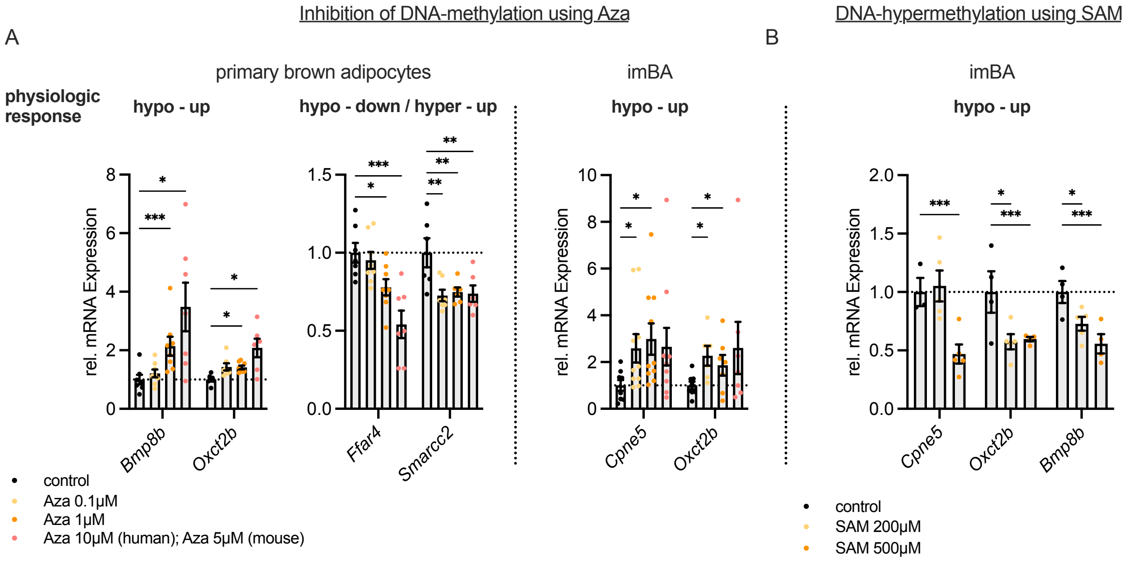

Supplementary Figure 5: *In vitro* validation of DNA methylation affecting expression of candidate genes. (A) Inhibition of DNA methylation using indicated concentrations of 5'aza-2'-deoxycytidine (Aza) in mouse immortalized (imBA, left) and primary brown adipocytes (right). Gene expression was analyzed by qPCR, N = 6-10 per condition. (B) Induction of DNA hypermethylation using indicated concentrations of S-adenosylmethionine (SAM) in mouse imBA. Gene expression was analyzed by qPCR, n = 3-5 per condition. Statistical significance was evaluated by two-way ANOVA with uncorrected Fischer's LSD. p <0.05 (*), p <0.01 (**), p <0.001 (***).

Supplementary Figure 6

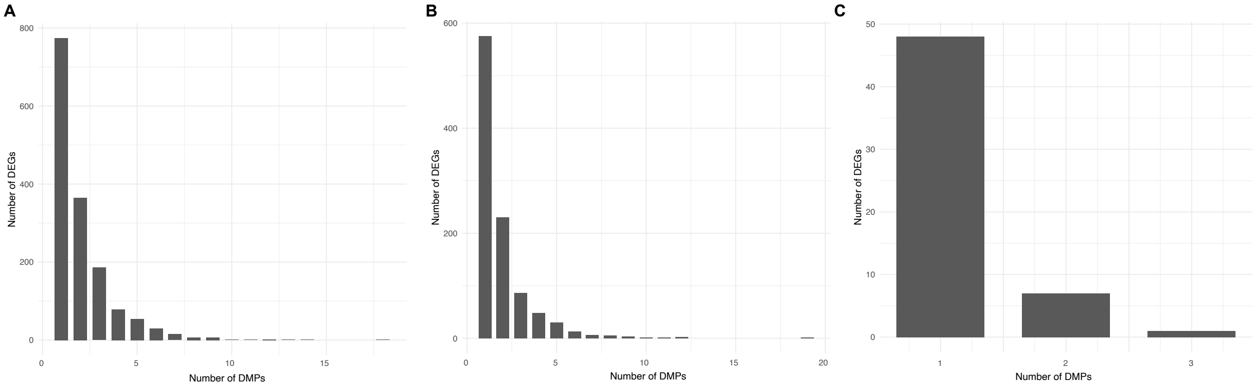

Supplementary Figure 6: Distribution of differentially methylated position associated with DMEGs. For each DEG within a DMEG, the number of corresponding differentially methylated position (DMPs) is shown for (A) $COLD_{lean}$, (B) $COLD_{DIO}$, and (C) $\Delta COLD$ condition pairs.

Supplementary Figure 7

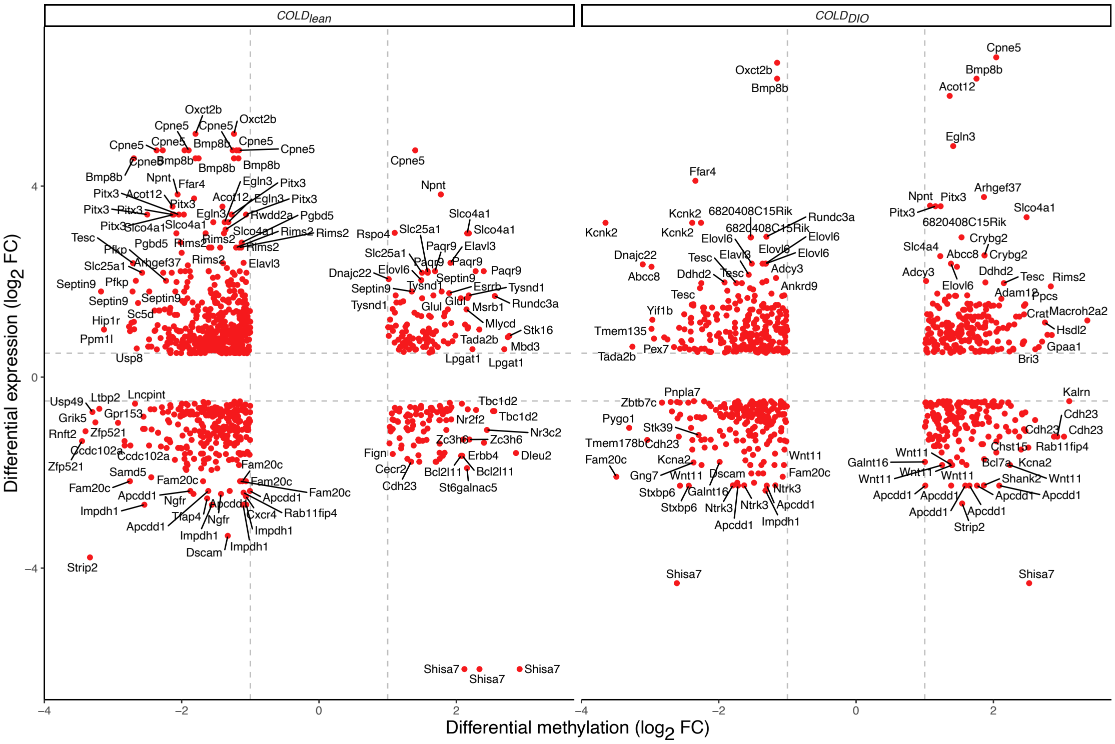

Supplementary Figure 7: Overlapping DMEGs between $COLD_{lean}$ and $COLD_{DIO}$. The scatter plot displays the $\log_2$ FC in gene expression and DNA methylation signals for all DMEGs which overlap in both $COLD_{lean}$ and $COLD_{DIO}$ condition. Colors correspond to highlights in Supplementary Figure 3. DMEGs with absolute $\log_2$ FC >0.5 in expression and >1 in methylation signals are shown, while DMEGs in the 5% and 95% quantile of both DMP and DEG $\log_2$ FC signals are labeled.

Supplementary Figure 8

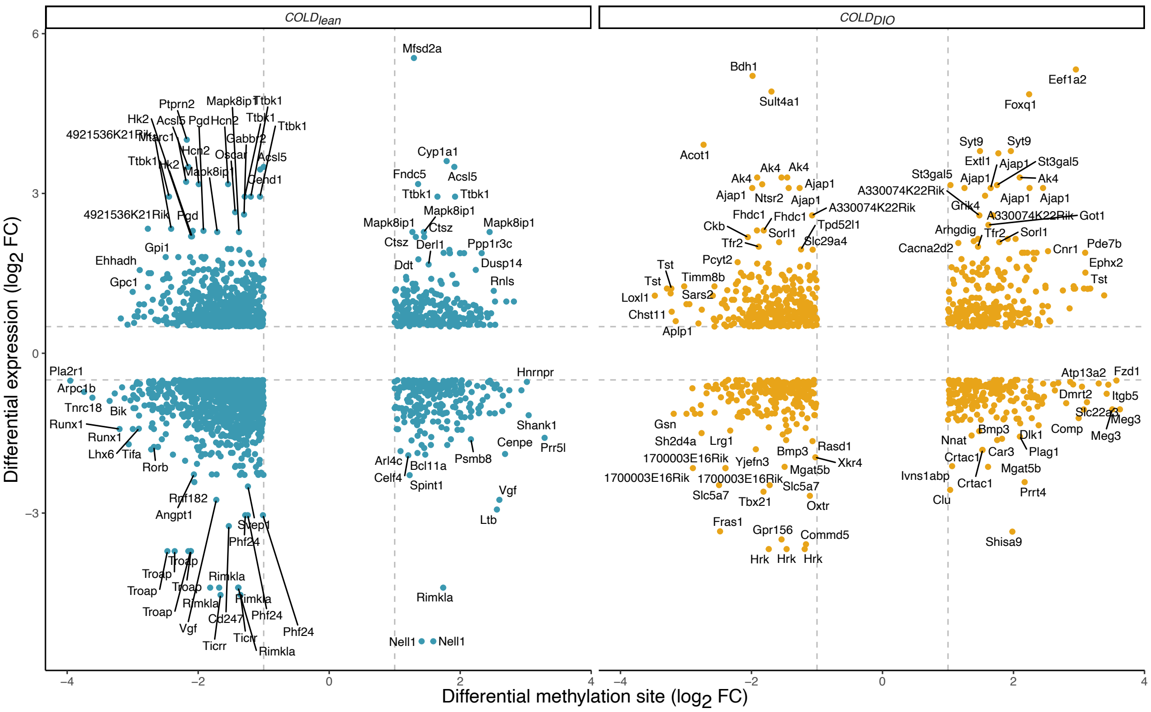

Suppl. Figure 8: DMEGs that are specifically regulated within each condition. The scatter plot displays the log_2 FC in gene expression and DNA methylation signals for all DMEGs which are specifically regulated in both $COLD_{lean}$ and $COLD_{DIO}$ conditions. Colors correspond to highlights in Supplementary Figure 3. DMEGs with absolute log_2 FC >0.5 in expression and >1 in methylation signals are shown, while DMEGs in the 5% and 95% quantile of both DMP and DEG log_2 FC signals are labeled.

Supplementary Table legends

Supplementary Table 1: Overview of primers used for PCR.

Supplementary Table 2: Overview of significant (FDR <0.05) DEGs for the $COLD_{lean}$ contrast. DEGs that overlap with Tayler et al. (2024) [1] (lean chow-fed mice, 8°C versus 28°C) are listed.

Supplementary Table 3: Overview of significant (FDR <0.05) DEGs for the $COLD_{DIO}$ contrast.

Supplementary Table 4: Overview of significant (FDR <0.05) DEGs for the *ΔCOLD* condition.

Supplementary Table 5: Overview of DMEGs in the $COLD_{lean}$ contrast with absolute expression log_2 FC >0.5 and absolute methylation log_2 FC >1, including the cell type assignment derived from single-cell RNAseq data obtained from [2].

Supplementary Table 6: Overview of DMEGs in the $COLD_{DIO}$ contrast with absolute expression $\log_2$ FC >0.5 and absolute methylation $\log_2$ FC >1.

Supplementary Table 7: Overview of DMEGs in the *ΔCOLD* contrast with absolute expression $\log_2$ FC >0.5 and absolute methylation $\log_2$ FC >1.

Supplementary Table 8: Significant Ingenuity Pathway Analysis (IPA) enrichment for canonical pathways categorized by four groups: those exhibiting similar regulation in both $COLD_{DIO}$ and $COLD_{lean}$ condition pairs, and those that are uniquely regulated in each individual condition (*ΔCOLD*, $COLD_{lean}$, and $COLD_{DIO}$.)

Supplementary Table 9: Significant IPA enrichment for upstream regulators categorized by four groups: those exhibiting similar regulation in both $COLD_{DIO}$ and $COLD_{lean}$ condition pairs, and those that are uniquely regulated in each individual condition.

Supplementary Table 10: Motif enrichment of transcription factor binding sites from JASPAR 2024 database within sequences in +-20nt windows of methylation loci of DMEGs similar regulated in both $COLD_{DIO}$ and $COLD_{lean}$ condition pairs, and those that are uniquely regulated in each individual condition. Random promoter sequences of equal length were sampled from mm10 reference genome as background. Similarly, sequences from uniquely regulated DMEGs in $COLD_{DIO}$ condition were enriched with sequences from $COLD_{lean}$ DMEGs as background with a one-sided hypergeometrix test. The table contains all motif enrichments with $p < 0.05$.

Supplementary References

[1] Taylor BC, Steinthal LH, Dias M, Yalamanchili HK, Ochsner SA, Zapata GE, et al. Histone proteoform analysis reveals epigenetic changes in adult mouse brown adipose tissue in response to cold stress. Epigenetics Chromatin 2024;17:12. https://doi.org/10.1186/s13072-024-00536-8.

[2] Shamsi F, Zheng R, Ho L-L, Chen K, Tseng Y-H. Comprehensive analysis of intercellular communication in the thermogenic adipose niche. Commun Biol 2023;6:761. https://doi.org/10.1038/s42003-023-05140-2.
